# Norepinephrine Induces Sertoli Cell Ferroptosis via Receptors Desensitization Causing Stress‐Related Male Reproductive Dysfunction

**DOI:** 10.1002/advs.202504817

**Published:** 2025-10-06

**Authors:** Lingyu Zhang, Shanfeng Gao, Xiaofan Xiong, Xin Liu, Rufeng Li, Xia Wang, Lin Han, Xuan Xiao, Xiaofei Wang, Wen Li, Yongxia Chang, Yuefeng Du, Juan Yang

**Affiliations:** ^1^ Department of Cell Biology and Genetics, School of Basic Medical Sciences Xi'an Jiaotong University Health Science Center Xi'an Shaanxi 710061 P. R. China; ^2^ Western China Science and Technology Innovation Port in precision medicine institute The Second Affiliated Hospital of Xi'an Jiaotong University Xi'an Shaanxi 710004 P. R. China; ^3^ Key Laboratory of Environment and Genes Related to Diseases (Xi'an Jiaotong University) Ministry of Education of China Xi'an Shaanxi 710061 P. R. China; ^4^ Department of Urology the First Affiliated Hospital of Xi'an Jiaotong University Xi'an Shaanxi 710061 P. R. China

**Keywords:** ferroptosis, male reproductive damage, norepinephrine, psychological stress, sertoli cells

## Abstract

Psychological stress poses a significant threat to male reproduction; however, the underlying molecular mechanisms remain poorly understood. Stress‐induced hyperactivation of the sympathetic nervous system triggers the secretion of norepinephrine (NE), a key mediator implicated in various pathophysiological processes. Although NE is linked to male reproductive dysfunction, the precise mechanism remains unclear. Here, it is demonstrated that psychological stress can induce Sertoli cell ferroptosis through NE, which is characterized by iron overload, lipid peroxidation, and altered expression of ferroptosis‐related proteins. Blockade of β‐adrenergic receptors (β‐ARs) with propranolol alleviated stress‐induced damage, inhibiting ferroptosis and promoting spermatogenesis. In vitro, selective β_1_‐ and β_2_‐AR antagonists reversed NE‐induced Sertoli cell ferroptosis. Mechanistically, NE activated β‐arrestin1, driving β‐ARs desensitization and internalization, which subsequently stimulated inhibitory G proteins (Gi), suppressed CREB1‐dependent GPX4 transcription, and promoted ferroptosis. The findings reveal NE‐induced β‐ARs desensitization as a mechanistic driver of Sertoli cell ferroptosis. β‐ARs signaling modulation is proposed as a potential therapeutic approach for alleviating stress‐associated male reproductive impairment.

## Graphical abstract

Psychological stress activates the sympathetic–adrenal axis, elevating norepinephrine (NE) and suppressing reproductive hormones, thereby impairing male reproduction. Excess NE overactivates and desensitizes β‐adrenergic receptors (β‐ARs), triggering Sertoli cell ferroptosis and disrupting spermatogenesis. This study identifies a novel NE–β‐ARs–ferroptosis axis linking stress to testicular injury, and shows that blocking β‐ARs signaling or ferroptosis mitigates the damage.

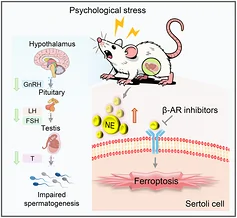

## 1 Introduction

Infertility affects ≈15% of married couples of reproductive age worldwide, with males accounting for ≈50% of these cases.^[^ ^1^ ^]^ Clinical studies indicated that psychological stress negatively impacts male fertility by disrupting semen parameters.^[^ ^2^, ^3^ ^]^ Adverse psychological factors contribute to reduced testosterone (T) levels and sperm quality.^[^ ^4^ ^]^ Therefore, psychological stress has emerged as a significant contributor to male reproductive impairment, warranting further investigation of its underlying mechanisms.

The sympathetic nervous system (SNS) serves as the body's primary rapid‐response network to psychological stress, with norepinephrine (NE) acting as its key effector molecule.^[^ ^5^, ^6^ ^]^ NE is released from sympathetic nerve terminals and adrenal chromaffin cells upon stress exposure, binding to β‐adrenergic receptors (β‐ARs) on target organs to orchestrate adaptive physiological changes. Classical β‐ARs signaling involves Gs protein‐coupled activation of adenylate cyclase, leading to accumulation of cyclic AMP (cAMP) and protein kinase A (PKA).^[^ ^7^, ^8^ ^]^ Sympathetic nerve fibers and catecholaminergic neuronal elements are distributed throughout the testes. NE regulates normal testicular physiology, but elevated NE levels have been linked to reduced testicular blood flow, increased oxidative stress, lower T levels, and impaired spermatogenesis.^[^ ^9^, ^10^ ^]^ Upregulation of β‐ARs leads to testicular inflammation and male infertility.^[^ ^11^ ^]^ However, whether elevated NE levels due to psychological stress contribute to male reproductive dysfunction.

Sertoli cells, somatic cells within the seminiferous epithelium,^[^ ^12^ ^]^ play an essential role in testicular development and function. Tight junctions formed between Sertoli cells constitute the blood‐testis barrier (BTB), creating a microenvironment necessary for spermatogenic differentiation and self‐renewal of spermatogonial stem cells.^[^ ^13^, ^14^ ^]^ Previous research has shown that early maternal separation in mice reduces the number of Sertoli and spermatogenic cells.^[^ ^15^ ^]^ Additionally, psychological stress compromises the integrity of BTB.^[^ ^16^ ^]^ However, the specific effects of psychological stress on the Sertoli cells remain poorly understood.

Ferroptosis is an iron‐dependent cell death distinct from apoptosis, necrosis, and autophagy. The core mechanisms of ferroptosis include iron overload, lipid peroxidation, and dysregulation of the antioxidant system, which have been confirmed to be closely related to male reproductive dysfunction.^[^ ^17^, ^18^ ^]^ Iron homeostasis is essential for spermatogenesis. Animal models have demonstrated that exposure to plasticizer, cadmium, busulfan, and obesity can induce testicular damage via ferroptosis.^[^ ^19^, ^20^, ^21^, ^22^ ^]^ Ferroptosis has been implicated in the pathophysiological processes of various psychological stress‐induced disorders, ranging from depression to heart disease.^[^ ^23^, ^24^, ^25^ ^]^ However, the role of ferroptosis in psychological stress‐induced male reproductive dysfunction remains unexplored.

In the present study, we demonstrated that psychological stress‐induced elevation of the stress hormone, NE, directly triggered ferroptosis in Sertoli cells. Using a four‐cohort experimental design (Saline, NE injection, Stress + Saline, Stress + Propranolol), we found that the NE injection was consistent with the stress phenotype. In contrast, propranolol effectively reversed stress‐induced ferroptosis and reproductive dysfunction. Both in vitro and in vivo experiments revealed that chronic NE activation of β‐ARs led to desensitization, resulting in decreased CREB1 and GPX4 expression and subsequent ferroptosis in Sertoli cells. Taken together, these findings provide insights into the mechanisms underlying stress‐induced testicular injury and suggest potential therapeutic strategies for reproductive dysfunction in stressed individuals.

## 2 Results

### 2.1 Psychological Stress Induces Reproductive Damage in Male Rats

**Figure 1:**
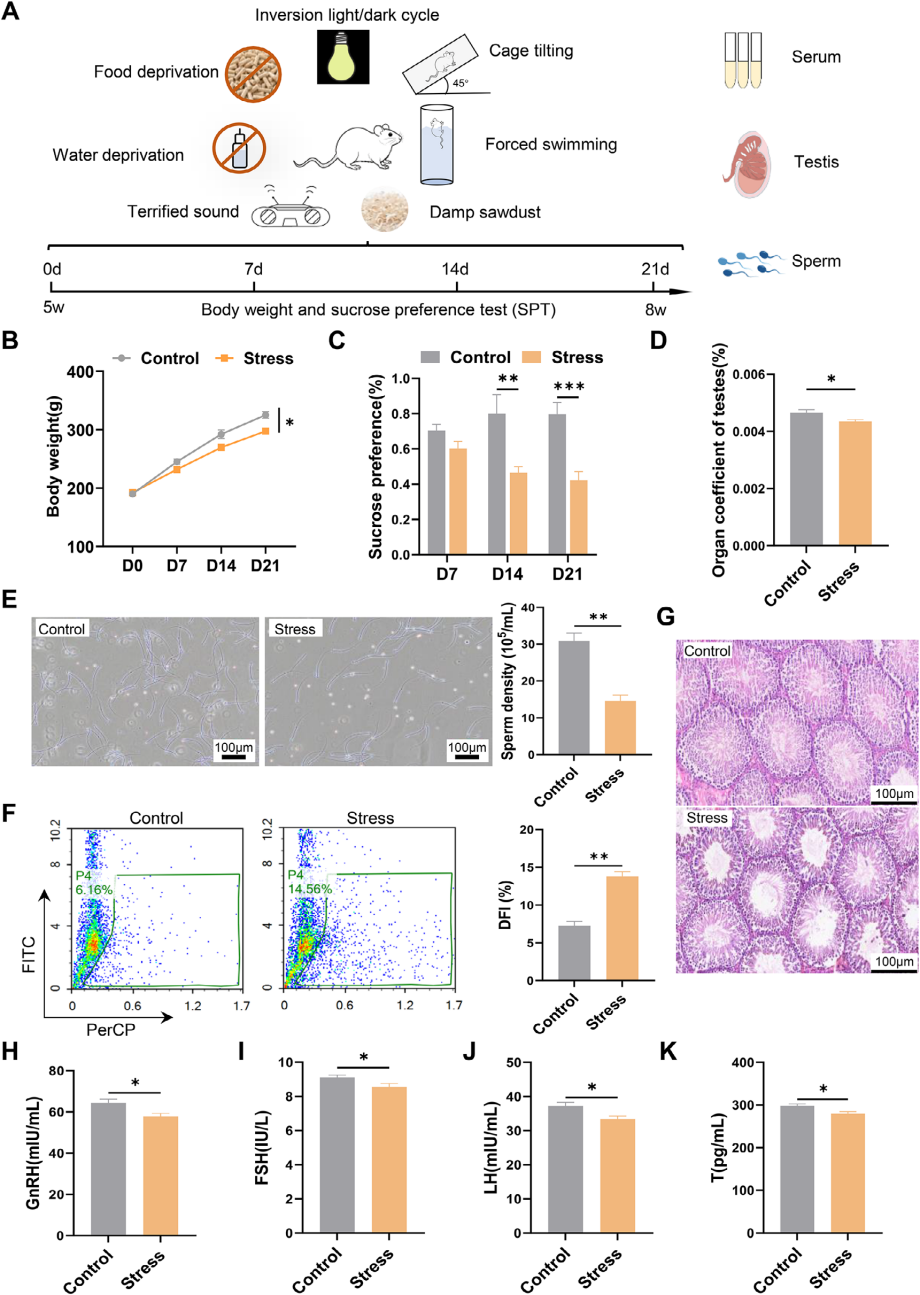
Psychological stress impairs male reproduction. A) Experimental timeline and sample collection. B) Body weight of control and stressed rats (n = 10). C) Sucrose preference test comparing stressed and control rats (n = 10). D) Testes organ coefficient in control and stress groups (n = 10). E) Representative images showing sperm density in the cauda epididymis (scale bar, 100 µm; n = 5). F) Sperm DNA fragmentation levels (n = 3). G) H&E staining of testicular tissue in control and stressed rats (scale bar, 100 µm). H–K) Serum levels of gonadotropin‐releasing hormone (GnRH), follicle‐stimulating hormone (FSH), luteinizing hormone (LH), and testosterone (T) in control and stressed rats (n = 10). Data are presented as mean ± SEM. Statistical analysis is performed using two‐way ANOVA or an unpaired two‐tailed Student's *t*‐test. ^*^ *p* < 0.05, ^**^ *p* < 0.01, ^***^ *p* < 0.001.

To investigate the effects of chronic psychological stress on male reproductive pathophysiology, we established a rat model using chronic unpredictable mild stress (CUMS) coupled with social isolation for 21 days (**Figure** **1**A). Stress exposure significantly impaired body weight, reduced sucrose preference, and activated the hypothalamic‐pituitary‐adrenal (HPA) axis (Figure 1B,C; Figure S1A–D, Supporting Information), indicating depression‐like phenotypes. Stress‐induced reproductive impairment was evidenced by a decrease in the organ coefficient of the testes, as well as histopathological alterations in the seminiferous tubules (Figure 1D,G; Figure S1E–H, Supporting Information). Furthermore, stress exposure reduced sperm density and increased DNA fragmentation (Figure 1E,F), accompanied by decreased serum levels of gonadotropin‐releasing hormone (GnRH), follicle‐stimulating hormone (FSH), luteinizing hormone (LH), and T (Figure 1H–K). These findings demonstrate that psychological stress damages reproduction in males.

### 2.2 Sertoli Cell Ferroptosis Mediates Stress‐Induced Decreased Spermatogenesis

**Figure 2:**
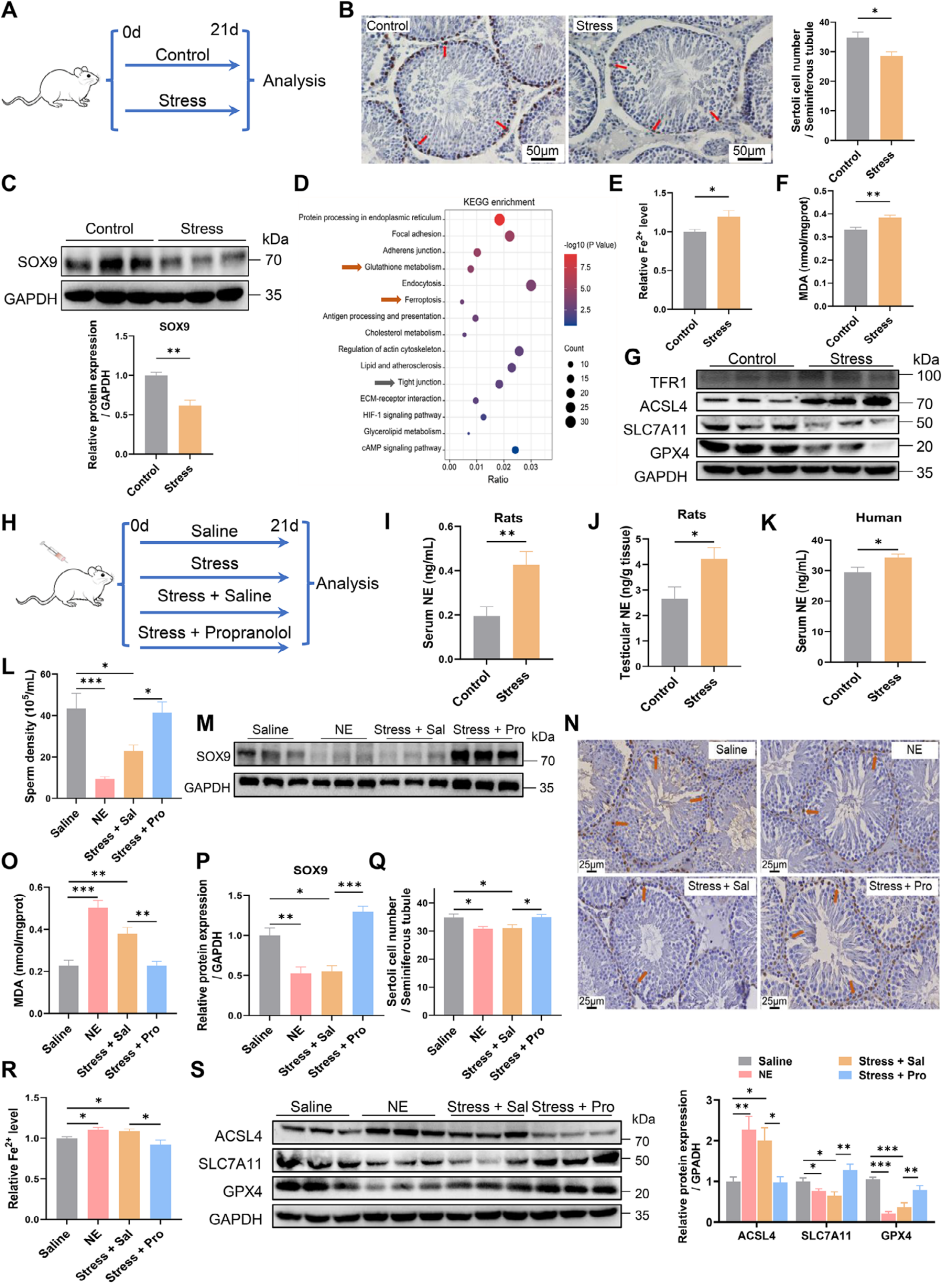
Psychological stress triggers Sertoli cell ferroptosis via NE‐mediated β‐adrenergic receptor activation. A) Schematic illustration of the experimental design (control vs stress group). B) WT1‐labeled Sertoli cells (red arrows) within the seminiferous tubule lumen (scale bar, 50 µm, n = 8). Quantification was based on 10 randomly selected tubules per animal. C) Western blot analysis of SOX9 expression in testicular tissue (n = 3). D) KEGG pathway enrichment analysis of scRNA‐seq data from clustered Sertoli cells (n = 3). E,F) Quantification of ferrous ion (Fe^2^⁺) and malondialdehyde (MDA) levels in the testes (n = 10). G) Western blot analysis of ferroptosis‐associated proteins (n = 3). H) Schematic of experimental design with added NE and propranolol treatments. I,J), NE levels in the serum and testis of control and stressed rats (n = 8). K) Serum NE levels in healthy controls (n = 31) and infertile men with psychological stress (n = 66). L) Quantitative of sperm density in the cauda epididymis (n = 5). M) Western blot of SOX9 expression across four experimental groups (n = 3). N) WT1‐labeled Sertoli cells in the seminiferous tubules (red arrows) (scale bar, 25 µm). O) MDA levels in the testes of four groups (n = 8). P) Quantitation of SOX9 in the testes (n = 3). Q) Quantification of WT1‐positive Sertoli cells per tubule (n = 8). R) Fe^2^⁺ levels in the testes of four groups (n = 8). S) Western blot of ACSL4, SLC7A11, and GPX4 in testes (n = 6). Data are presented as mean ± SEM. Statistical significance was assessed by unpaired two‐tailed Student's *t*‐test for two‐group comparisons, or one‐way ANOVA followed by Holm–Šídák's post hoc test for multiple groups. ^*^ *p* < 0.05; ^**^ *p* < 0.01; ^***^ *p* < 0.001.

Sertoli cells are somatic cells that support spermatogenesis and provide structural and metabolic assistance for germ cell development through tightly regulated hormonal interactions.^[^ ^26^ ^]^ Since each Sertoli cell sustains a finite germ cell population, their numerical reduction directly compromises spermatogenic capacity.^[^ ^27^ ^]^ Immunohistochemical analysis and western blotting revealed a significant decrease in Sertoli cell markers in the testes subjected to stress, suggesting cell loss (**Figure** **2**A–C). Given that Sertoli cells undergo terminal differentiation after puberty in rodents,^[^ ^28^ ^]^ this reduction reflects stress‐triggered cell death rather than impaired proliferation.

To explore the mechanism of Sertoli cell loss, we compared the control and psychological stress groups by clustering Sertoli cells using previously generated single‐cell RNA sequencing (scRNA‐seq) data.^[^ ^29^ ^]^ Although the KEGG analysis identified multiple enriched pathways, our study aimed to elucidate how psychological stress reduces the number of Sertoli cells and impairs their function, ultimately disrupting spermatogenesis. Although the ferroptosis and glutathione metabolism pathways were not among the top‐ranked pathways regarding the enrichment score, they were significantly enriched in the stress group (Figure 2D) and, more importantly, closely aligned with our biological hypothesis. Ferroptosis, a form of iron‐dependent programmed cell death, has been implicated in Sertoli cell injury and male reproductive disorders.^[^ ^30^ ^]^ This was confirmed by gene ontology (GO) analysis, which showed significant enrichment in the biological processes associated with glutathione metabolism, cellular responses to metal ions, lipid transport, and oxidative stress (Figure S2A, Supporting Information). In particular, we observed a dramatic decrease in the levels of glutathione peroxidase 4 (GPX4), a critical ferroptosis regulator, in Sertoli cells under stress conditions (Figure S2B, Supporting Information). KEGG analysis also revealed enrichment of pathways related to tight junction organization, prompting us to examine further the integrity of the BTB (Figure 2D). Concomitantly, stress also disrupted the integrity of the BTB, as evidenced by the markedly reduced expression of the tight junction proteins ZO‐1 and Claudin‐11(Figure S2C, Supporting Information). These results suggest that psychological stress induces ferroptosis in Sertoli cells, impairing the tight junction.

To further validate the scRNA‐seq results, we assessed ferrous ion levels, lipid peroxidation, and ferroptosis‐related proteins in testicular tissue. Ferroptosis is characterized by ferrous iron overload and the accumulation of lipid peroxides such as malondialdehyde (MDA), a biomarker of lipid peroxidation.^[^ ^31^, ^32^ ^]^ Both ferrous iron accumulation and lipid peroxidation were increased in the testes of stressed rats (Figure 2E,F). We also observed reduced levels of GPX4 following psychological stress, as detected by western blot. Additionally, solute carrier family 7 member 11 (SLC7A11), a cystine/glutamate antiporter involved in glutathione synthesis, was detected and found to be downregulated in the testes of the stress group, as shown by western blot. Acyl‐CoA synthetase long‐chain family member 4 (ACSL4), a widely used marker of ferroptosis, was elevated in stress‐treated testes. No significant differences in transferrin receptors (TFR1) between the two groups (Figure 2G; Figure S2D, Supporting Information). To further validate that stress‐induced ferroptosis in Sertoli cells contributes to impaired spermatogenesis, we conducted animal experiments using a ferroptosis inhibitor (Ferrostain‐1, Fer‐1) with stress exposure. Notably, treatment with Fer‐1 significantly preserved sperm density and maintained Sertoli cells' number and functional markers (Figure S3A–C, Supporting Information). Moreover, ferroptosis‐related indicators, including MDA, ferrous ions, and ferroptosis‐related proteins, were ameliorated in the testes compared with the stress group (Figure S3D–F, Supporting Information). These findings suggest that psychological stress impairs spermatogenesis via Sertoli cell ferroptosis.

### 2.3 Norepinephrine Mediates Stress‐Induced Sertoli Cell Ferroptosis via β‐Adrenergic Receptor Activation

Given the critical role of NE and β‐ARs in regulating stress responses and testicular function,^[^ ^33^ ^]^ we hypothesized that NE might mediate stress‐induced ferroptosis of Sertoli cells. Our data revealed a significant increase in NE levels in both the testes and the serum of male rats subjected to stress (Figure 2I,J). Similarly, serum NE levels were elevated in infertile patients experiencing psychological stress (Figure 2K). Furthermore, Spearman's correlation analysis of the human cohort demonstrated a significant negative correlation between psychological stress and sperm quality parameters. Specifically, patients with psychological stress exhibited lower percentages of forward‐moving spermatozoa, reduced total sperm concentration and count, decreased rates of normal morphology, and diminished total sperm vitality (Figure S2E and Table S1, Supporting Information). To further clarify the role of NE, we conducted experiments involving NE injection, psychological stress with and without the β‐ARs inhibitor (Propranolol) (Figure 2H). Our findings indicated that NE exposure led to slower body weight gain, reduced sucrose preference, damaged seminiferous tubules, and decreased the testis organ coefficient and sperm density, consistent with psychological stress's effects. Propranolol significantly attenuated these stress‐induced adverse effects (Figure 2L; Figure S4A–E, Supporting Information).

To further investigate whether NE is involved in stress‐induced Sertoli cell ferroptosis, we examined the Sertoli cell number and ferroptosis‐related indicators. Consistent with psychological stress, NE exposure significantly reduced the Sertoli cell count, elevated lipid peroxidation, and increased ferrous ion levels in the testes (Figure 2M–R). Western blot analysis showed downregulation of GPX4 and SLC7A11 and upregulation of ACSL4 in both the NE and stress groups (Figure 2S). A marked reduction in ZO‐1 and Claudin‐11 expression indicated impaired Sertoli cell function in both NE‐treated and stress‐exposed groups (Figure S4F, Supporting Information). Propranolol effectively prevented stress‐induced ferroptosis and Sertoli cell dysfunction. These data suggest elevated NE levels resulting from stress contribute to Sertoli cell ferroptosis through β‐ARs activation.

### 2.4 Norepinephrine Induces Ferroptosis in TM4 Cells

**Figure 3:**
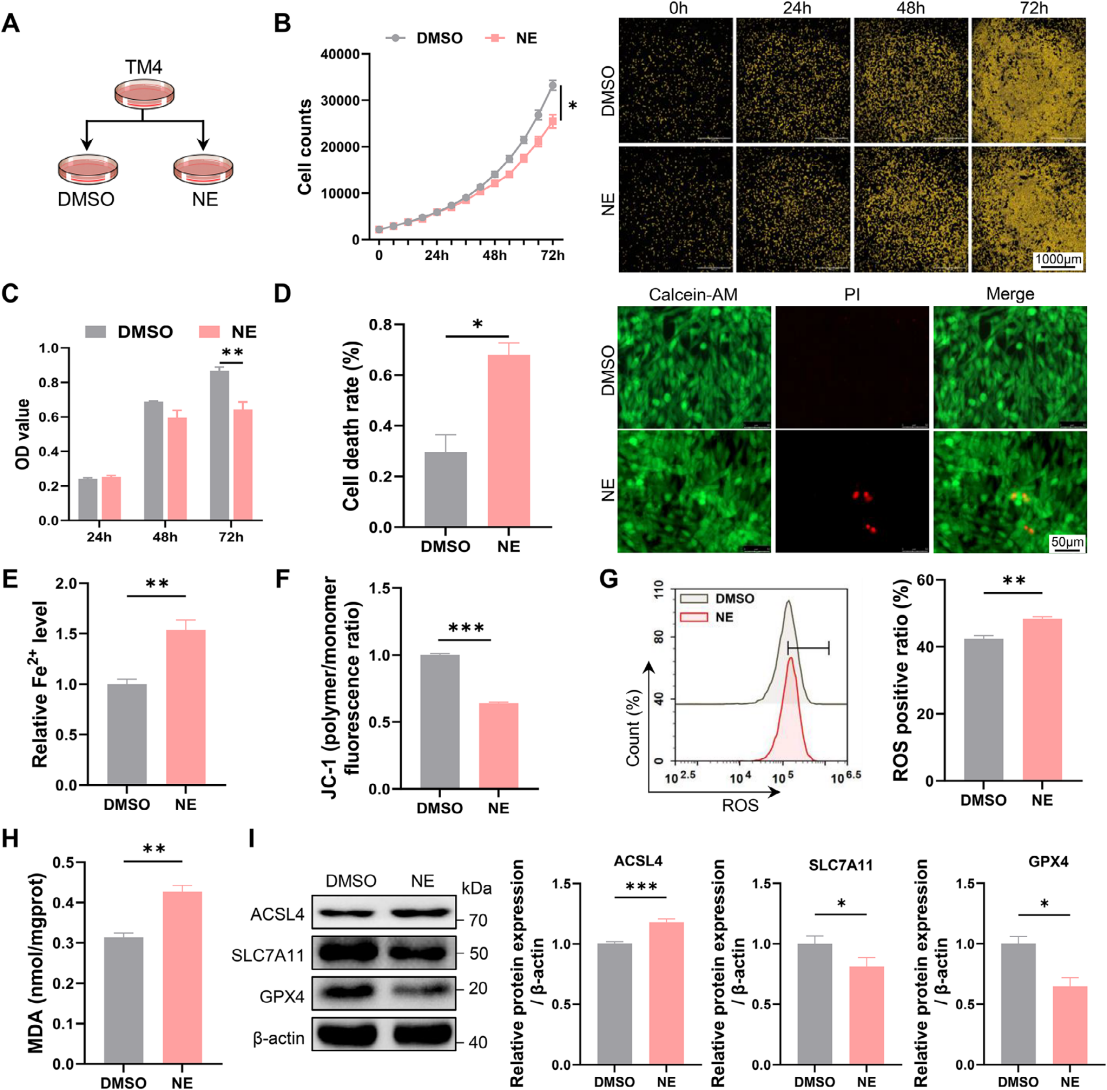
NE induces ferroptosis in TM4 cells. A) Experimental grouping of TM4 cells treated with NE or DMSO. B) Cell counts assessed by live cell imaging (scale bar, 1000 µm, n = 4). C) Cell viability in the DMSO and NE group (n = 3). D) Calcein‐AM/PI staining after 48 h NE exposure (scale bars, 50 µm, n = 4). E) Intracellular Fe^2^⁺ content following NE treatment (n = 3). F) Mitochondrial membrane potential after NE exposure (n = 3). G) ROS levels after NE exposure (n = 3). H) MDA levels after NE exposure (n = 3). I) Western blot and quantification of ACSL4, SLC7A11, and GPX4 after NE treatment (n = 3). Data are shown as mean ± SEM. Statistical analysis was performed by unpaired two‐tailed Student's *t*‐test or two‐way ANOVA. ^*^ *p* < 0.05; ^**^ *p* < 0.01; ^***^ *p*< 0.001.

To investigate the molecular mechanisms underlying NE‐induced ferroptosis in Sertoli cells, a series of experiments was conducted using the TM4 Sertoli cell line (**Figure** **3**A). We found a time‐dependent decline in cell count and viability following NE exposure, as quantified by live‐cell analysis and MTT assay (Figure 3B,C). Quantitative fluorescence microscopy analysis of calcein‐AM/ Propidium Iodide (PI) dual staining revealed increased PI‐positive cells following NE treatment, indicating that NE promotes cell death (Figure 3D). Notably, intracellular ferrous ion accumulation was markedly enhanced in NE‐treated cells, while the mitochondrial membrane potential was substantially diminished, indicating mitochondrial dysfunction (Figure 3E,F; Figure S5A, Supporting Information). Additionally, we observed a significant increase in ROS and MDA levels, indicating increased lipid peroxidation (Figure 3G,H; Figure S5B, Supporting Information). Western blot analysis further confirmed the characteristic ferroptosis‐related changes, including the downregulation of SLC7A11 and GPX4 and the upregulation of ACSL4 (Figure 3I). Immunofluorescence staining for ACSL4 and GPX4 further confirmed the expression of these ferroptosis markers at the cellular level, providing direct evidence of NE‐induced ferroptosis in Sertoli cells (Figure S5D,E, Supporting Information). Moreover, western blot analysis of ZO‐1 and Claudin‐11 indicated that NE exposure impaired the expression of Sertoli cell barrier‐associated proteins (Figure S5C, Supporting Information).

**Figure 4:**
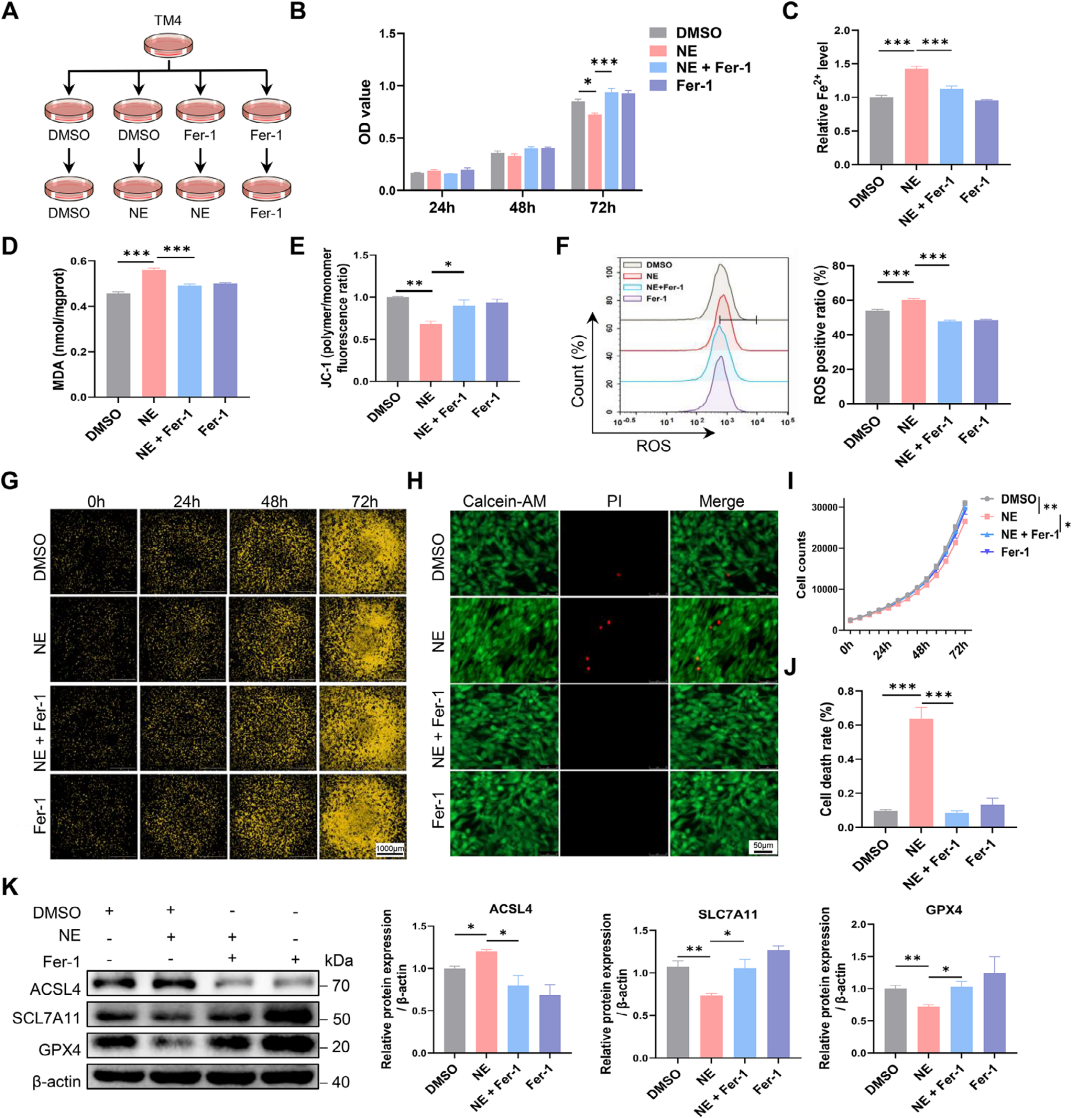
Fer‐1 rescues NE‐induced ferroptosis in TM4 cells. A) TM4 cells were treated with NE with or without Fer‐1 (0.5 µM). B) Cell viability was assayed using the MTT assay (n = 4). C) Fe^2+^ overload after NE exposure was reversed by Fer‐1 (n = 3). D) Lipid peroxidation induced by NE was reduced by Fer‐1 (n = 3). E) Mitochondrial membrane potential loss was restored by Fer‐1 (n = 3). F) Elevated ROS levels following NE exposure were suppressed by Fer‐1 (n = 3). G) Decreased cell counts after NE exposure were restored by Fer‐1. H) Calcein‐AM/PI staining showed reduced NE‐induced cell death with Fer‐1. I,J) Quantification of cell count (n = 3) and cell death ratio (n = 4). K) Western blot analysis showed reversal of ferroptosis‐related protein expression by Fer‐1 (n = 3–4). Data are presented as mean ± SEM. Statistical significance was assessed using one‐way ANOVA or two‐way ANOVA followed by Dunnett post hoc test. ^*^ *p* < 0.05; ^**^ *p* < 0.01; ^***^ *p* < 0.001.

To establish ferroptosis as the primary mechanism of action, we used Fer‐1 in our experimental design (**Figure** **4**A). Fer‐1 rescued the NE‐induced reduction in cellular viability and cell count (Figure 4B,G,I). Calcein‐AM/PI staining revealed a decrease in the proportion of dead cells with Fer‐1 co‐treatment compared to that in the NE‐only group (Figure 4H,J). Fer‐1 administration attenuated key ferroptosis hallmarks: ROS, MDA, ferrous ions and mitochondrial membrane potential recovered to control levels (Figure 4C–F; Figure S6A,B, Supporting Information). Western blot analysis demonstrated that Fer‐1 normalized the expression of ferroptosis regulators, rescuing the NE‐mediated suppression of SLC7A11 and GPX4 while reducing ACSL4 overexpression (Figure 4K). Furthermore, immunofluorescence staining showed that Fer‐1 reversed NE‐induced upregulation of ACSL4 and downregulation of GPX4 in Sertoli cells, providing more intuitive evidence for the involvement of ferroptosis (Figure S6D,E, Supporting Information). In addition, the expression of ZO‐1 and Claudin‐11 was restored by Fer‐1 treatment (Figure S6C, Supporting Information), indicating that ferroptosis contributes to Sertoli cell dysfunction. These findings provide compelling evidence that NE exposure triggers ferroptosis in Sertoli cells.

### 2.5 β_1_‐ and β_2_‐ Adrenergic Receptors Mediate Norepinephrine‐Induced Ferroptosis

**Figure 5:**
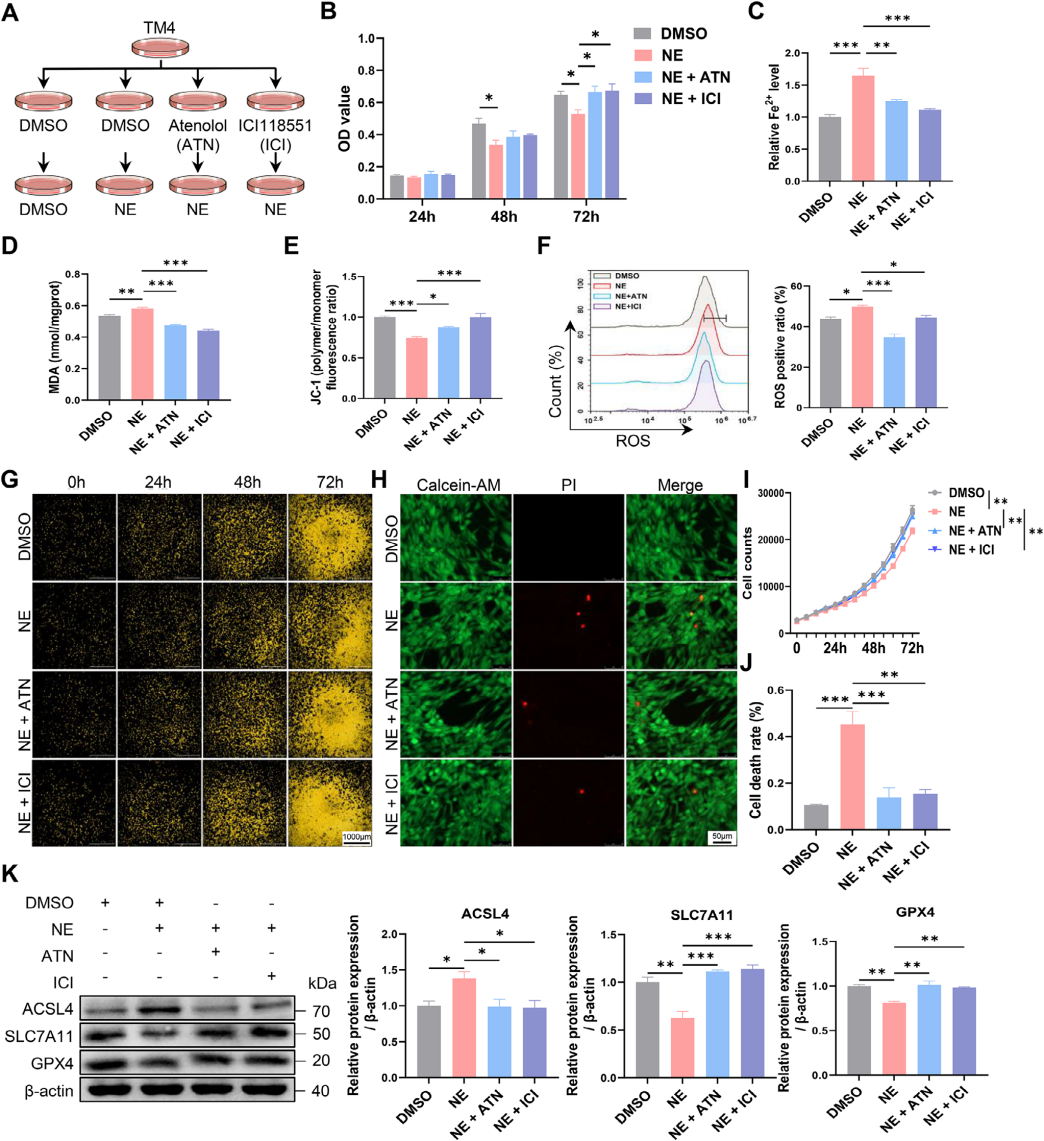
Inhibition of β‐ARs rescues NE‐induced ferroptosis. A) TM4 cells were treated with NE, Atenolol (ATN, β_1_‐AR specific, 1 µM), or ICI118551(ICI, β_2_‐AR specific, 1 µM). B) Atenolol or ICI118551 reversed the NE‐induced reduction in Sertoli cell viability (n = 4). C) Fe^2+^ overload induced by NE exposure was reversed by Atenolol or ICI118551 (n = 3). D) Increased lipid peroxidation following NE exposure was mitigated by Atenolol or ICI118551 (n = 3). E) Decreased mitochondrial membrane potential after NE exposure was restored by Atenolol or ICI118551 (n = 3). F) Increased ROS levels following NE treatment were suppressed by β‐ARs inhibition (n = 3). G) Decreased cell counts after NE exposure were restored by Atenolol or ICI118551. H) Calcein‐AM/PI staining showed that cell death due to NE exposure was abrogated using Atenolol or ICI118551. I,J) Quantification of viable cell number and cell death ratio after different treatments (n = 3). K) Western blot analysis of ACSL4, SLC7A11, and GPX4 expression in TM4 cell lysates from each treatment group (n = 3–4). Data are presented as mean ± SEM. Statistical significance was assessed using one‐way ANOVA or two‐way ANOVA followed by Dunnett post hoc test. ^*^ *p* < 0.05; ^**^ *p* < 0.01; ^***^ *p* < 0.001.

The vivo experiments demonstrated that inhibiting β‐ARs significantly alleviated male reproductive damage and reduced ferroptosis induced by psychological stress (Figure 2; Figure S4, Supporting Information). Both β_1_‐ and β_2_‐adrenergic receptor subtypes are expressed in Sertoli cells.^[^ ^34^ ^]^ To investigate the specific contributions of each subtype, we employed selective antagonists: Atenolol (ATN, β_1_‐AR specific) and ICI118551 (ICI, β_2_‐AR specific) (**Figure** **5**A). Both receptor blockers effectively rescued the NE‐induced decrease in Sertoli cell viability and cell count (Figure 5B,G,I). Inhibition of either β_1_‐ or β_2_‐AR alleviated the NE‐induced increase in cell death (Figure 5H,J). Receptor antagonism suppressed lipid peroxidation, attenuated ROS overproduction, and restored intracellular ferrous ion concentrations and mitochondrial membrane potentials (Figure 5C–F; Figure S7A,B, Supporting Information). Moreover, immunofluorescence staining demonstrated that ATN or ICI reversed the NE‐induced increase in ACSL4 expression and decreased in GPX4 expression (Figure S7D,E, Supporting Information). Meanwhile, blocking β_1_‐ or β_2_‐AR reversed NE‐induced alterations in the expression of SLC7A11, GPX4, and ACSL4 (Figure 5K). Blocking β_1_‐ or β_2_‐AR normalized tight junction markers ZO‐1 and Claudin‐11 protein levels, restoring Sertoli cell barrier function (Figure S7C, Supporting Information). These results suggest that β_1_‐ and β_2_‐AR cooperatively mediate NE‐induced ferroptosis signaling in Sertoli cells.

### 2.6 Norepinephrine Inhibits CREB1 and GPX4 Expression Through β‐Adrenergic Receptor Desensitization

**Figure 6:**
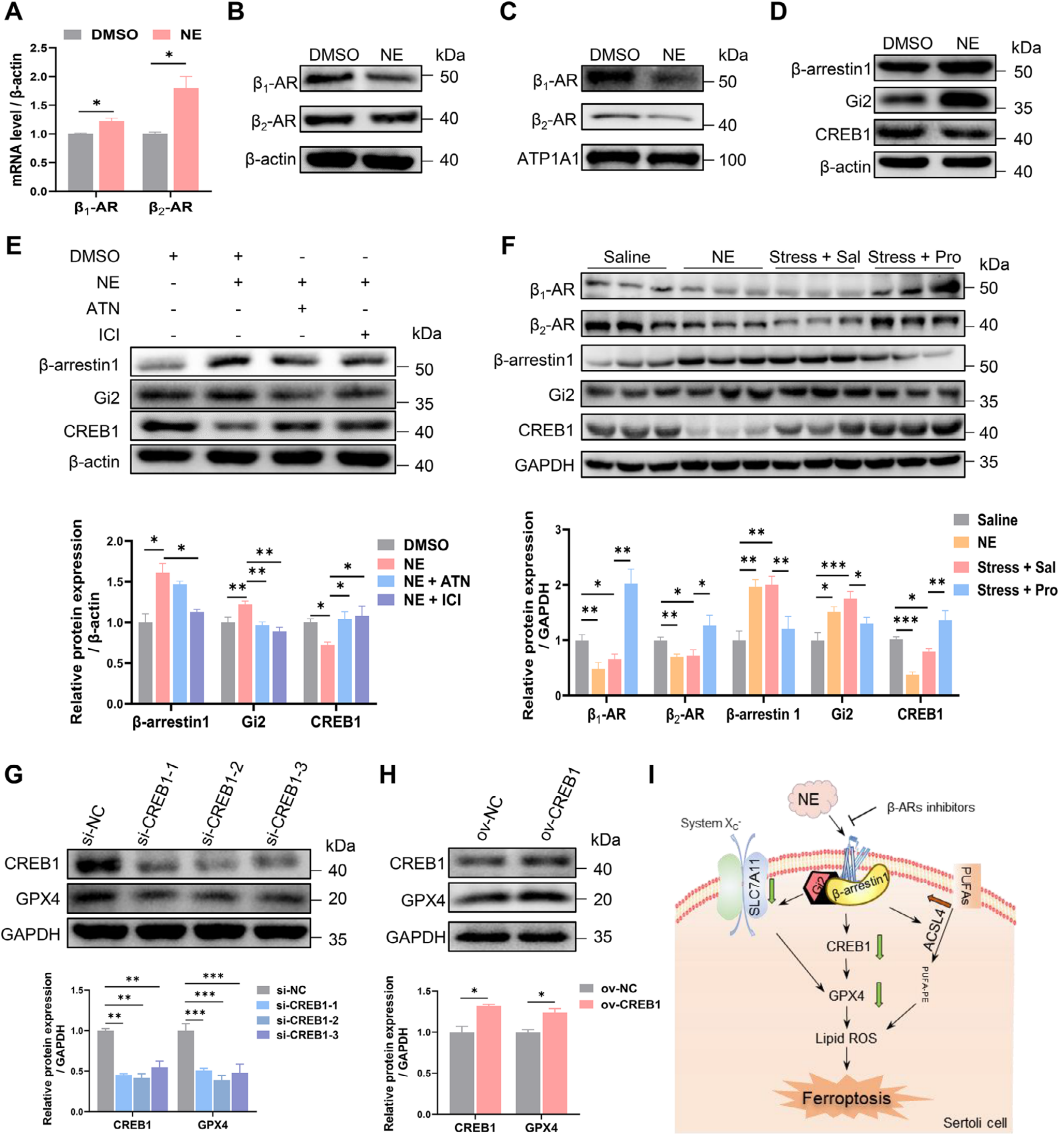
NE induces Sertoli cell ferroptosis through β‐ARs desensitization and CREB1/GPX4 suppression. A) NE increased transcription of β_1_‐AR and β_2_‐AR (n = 3). B) Total β_1_‐AR and β_2_‐AR protein levels decreased after NE exposure. C) Membrane β_1_‐AR and β_2_‐AR protein levels were reduced following NE exposure. D) NE increased β‐arrestin1 and Gi2 levels and inhibited CREB1 protein levels. E) Atenolol or ICI 118 551 reversed NE‐induced changes in β‐arrestin1, Gi2, and CREB1 protein levels (n = 4). F) Western blot and quantification of β_1_‐AR, β_2_‐AR, β‐arrestin1, Gi2, and CREB1 expression in testicular tissues (n = 6). G) Western blot analysis of CREB1 and GPX4 protein levels after siCREB1 knockdown (n = 3). H) CREB1 over expression enhanced CREB1 and GPX4 protein levels (n = 3). I) Mechanism of NE‐induced ferroptosis in Sertoli cells via β‐ARs overactivation. Data are expressed as mean ± SEM. For comparisons among multiple groups, one‐way ANOVA followed by Holm–Šídák's or Dunnett post hoc test. For comparisons between two groups, an unpaired two‐tailed Student's *t*‐test was used. ^*^ *p* < 0.05; ^**^ *p* < 0.01; ^***^ *p* < 0.001.

The canonical NE signaling cascade involves β‐ARs activation, which triggers cAMP‐dependent pathways that regulate CREB1 transcriptional activity.^[^ ^35^, ^36^ ^]^ Although NE exposure upregulated β_1_‐ and β_2_‐AR mRNA levels (**Figure** **6**A), western blot revealed paradoxical reductions in total receptor protein and membrane‐localized receptors (Figure 6B,C; Figure S8A,B, Supporting Information), accompanied by downregulation of CREB1 protein (Figure 6D; Figure S9A, Supporting Information). Since β‐ARs desensitization attenuates cAMP in Sertoli cell,^[^ ^37^ ^]^ we hypothesized that prolonged receptor activation drives β‐ARs desensitization and internalization, resulting in CREB1 downregulation.

To further elucidate the temporal dynamics of β‐ARs expression and ferroptosis‐related protein regulation upon NE stimulation, we performed time‐course analyses of membrane and total proteins at 0, 12, 24, 36, and 48 h. Membrane β_1_‐AR protein gradually increased between 12 and 36 h, showing significant elevation at 24 and 36 h compared to the control, but markedly declined at 48 h. β_2_‐AR membrane protein increased from 12 to 24 h, peaking at 24 h, then decreased to control levels at 36 h and significantly dropped at 48 h (Figure S8D, Supporting Information). Dynamic changes in ferroptosis‐associated proteins, ACSL4 levels were significantly elevated at 12 and 24 h, returned to baseline at 36 h, and increased at 48 h. In contrast, SLC7A11 and GPX4 were downregulated considerably at 48 h, with no marked trends observed at earlier time points (Figure S8C, Supporting Information). These findings suggest complex temporal regulation of ferroptosis markers during NE exposure. To further explore the intracellular trafficking and fate of β‐ARs upon NE stimulation, we conducted immunofluorescence co‐localization analysis with EEA1 (early endosome) and LAMP1 (lysosome). Mander's overlap coefficient (M2) quantified the degree of co‐localization, which reflects the proportion of β‐ARs signal overlapping with each organelle marker. NE treatment promoted receptor internalization, as evidenced by increased co‐localization of β‐ARs with EEA1 at 24 h (Figure S10A,B, Supporting Information). By 48 h, β‐ARs showed enhanced co‐localization with LAMP1, suggesting lysosomal targeting and degradation (Figure S10C,D, Supporting Information). This process coincided with a marked reduction in total β‐ARs protein levels (Figure 6B; Figure S8A, Supporting Information), indicating that prolonged NE exposure induces receptor desensitization and degradation via the endosome–lysosome pathway.

β‐arrestin1, a critical regulator of G protein‐coupled receptor (GPCR) desensitization and signaling,^[^ ^38^ ^]^ was upregulated after NE exposure (Figure 6D; Figure S9A, Supporting Information). Furthermore, NE enhanced the transcription of inhibitory G proteins (Gi), particularly Gi2 (Figure S9B, Supporting Information), which was confirmed at the protein level (Figure 6D; Figure S9A, Supporting Information). The suppressive effect of Gi‐coupled receptor signaling on CREB1 activation has been well established; Gi activation inhibits adenylyl cyclase activity, reduces intracellular cAMP levels, and consequently downregulates the PKA/CREB signaling cascade,^[^ ^39^, ^40^ ^]^ providing a mechanistic basis for our observations. We conducted siRNA‐mediated knockdown experiments to validate further the role of β‐arrestin1 and Gi2 in mediating CREB1 suppression and ferroptosis signaling. Knockdown of β‐arrestin1 reversed the NE‐induced upregulation of both β‐arrestin1 and Gi2, and restored the expression of CREB1 as well as ferroptosis‐related markers ACSL4, SLC7A11, and GPX4 (Figure S11A–C, Supporting Information). Similarly, Gi2 knockdown effectively suppressed NE‐induced Gi2 and ACSL4 expression while rescuing CREB1, SLC7A11, and GPX4 levels (Figure S11D–F, Supporting Information). These results underscore the critical roles of β‐arrestin1 and Gi2 in β‐ARs overactivation‐driven CREB1 inhibition and ferroptosis. Furthermore, blockade of β‐ARs with Atenolol or ICI118551 reversed NE‐induced desensitization and CREB1 downregulation (Figure 6E). In vivo validation demonstrated that stress‐induced β‐ARs and CREB1 downregulation with concomitant β‐arrestin1 and Gi2 elevations through the overactivation of β‐ARs by NE (Figure 6F). CREB1 is a transcriptional regulator of GPX4, and its knockdown triggers ferroptosis.^[^ ^41^ ^]^ We found that *CREB1* knockdown reduced GPX4 mRNA and protein levels without altering ACSL4 or SLC7A11 levels in Sertoli cells (Figure 6G; Figure S12A,B, Supporting Information). Conversely, *CREB1* overexpression increased GPX4 expression (Figure 6H; Figure S12C, Supporting Information). These results suggest that elevated NE induces β‐arrestin1‐mediated β‐ARs desensitization and Gi2 upregulation, suppressing CREB1‐dependent GPX4 transcription and causing ferroptosis in Sertoli cells.

## 3 Discussion

Emerging evidence indicates that psychological stress disrupts the endocrine system, adversely affecting male reproductive health.^[^ ^42^, ^43^ ^]^ Although stress activates multiple hormonal pathways, including the HPA axis and the sympathetic nervous system, the specific role of NE in reproductive dysfunction remains poorly understood. Our study identified β‐ARs overactivation, driven by elevated NE levels, as a key mechanism for stress‐induced ferroptosis in Sertoli cells. This overactivation leads to receptor desensitization and the upregulation of Gi, which suppresses CREB1‐dependent GPX4 expression and results in Sertoli cell ferroptosis (Figure 6I). Although TM4 cells are widely used for Sertoli cell studies because of their ease of manipulation, they are derived from immature mouse Sertoli cells. They may not fully represent adult rat Sertoli cell physiology. However, future studies using primary rat Sertoli cells are warranted to confirm these findings. Although 5–6‐week‐old SD rats are not fully mature during spermatogenesis, this age was chosen to model the effects of early stress on Sertoli cell function before full spermatogenic maturity, allowing us to capture early cellular dysfunction.

In our study, psychological stress elevated serum and testicular NE levels are consistent with reports of persistent NE elevation during chronic stress.^[^ ^44^ ^]^ The NE elevation‐induced ferroptosis in Sertoli cells aligns with previous evidence linking catecholamines to redox imbalance in peripheral tissues.^[^ ^45^, ^46^, ^47^ ^]^ Psychological stress is known to compromise testicular antioxidant defenses,^[^ ^29^, ^48^ ^]^ and redox imbalance has been shown to affect sexual behavior and fertility negatively.^[^ ^49^ ^]^ Our data demonstrate that stress‐induced elevation of NE exacerbates intracellular oxidative stress in Sertoli cells. Mitochondria, central regulators of iron metabolism and major ROS sources, contribute to oxidative stress and ferroptosis in cells when mitochondrial membrane potential is disrupted.^[^ ^50^, ^51^ ^]^ Previous studies have established that lipid peroxidation‐induced ferroptosis in Sertoli cells contributes to male reproductive disorders.^[^ ^21^, ^52^, ^53^, ^54^, ^55^ ^]^ We demonstrate that stress‐induced elevation of NE triggers ferroptosis in Sertoli cells, ultimately impairing male reproduction. Furthermore, the potential interaction between NE and other stress‐related hormones, such as cortisol, deserves further exploration, as cortisol has been reported to disrupt mitochondrial function and redox homeostasis in Sertoli cells, which are critically linked to ferroptotic vulnerability.^[^ ^56^ ^]^ Thus, exploring the combined effects of NE and cortisol on the ferroptotic pathways may provide deeper insights into the neuroendocrine regulation of male reproductive dysfunction.

The discovery that β‐ARs desensitization is a central mechanism driving NE‐induced ferroptosis in Sertoli cells reveals a previously unrecognized link between neuroendocrine stress responses and the male reproductive system. Both β_1_‐ and β_2_‐AR subtypes mediated NE‐triggered ferroptosis. Our time‐course analysis suggested that β‐ARs internalization and degradation precede or coincide with changes in ferroptosis‐related proteins in Sertoli cells. Upon receptor activation, β‐ARs internalization occurred alongside a rapid increase in ACSL4, promoting lipid peroxidation. Ferroptosis is aggravated as the antioxidant defenses mediated by SLC7A11 and GPX4 decline. Although β‐ARs desensitization is a protective adaptation during acute stress, chronic sustained activation drives disease progression through receptor internalization. Desensitization of β‐ARs has been associated with the onset and progression of various diseases, including heart failure, asthma, obesity, and neurodegenerative diseases.^[^ ^7^, ^57^ ^]^ Our study further demonstrated that β‐ARs desensitization played a significant role in impaired male reproductive health. Though we found that the successful rescue of spermatogenesis by propranolol validated β‐ARs blockade as a therapeutic strategy in rats, interspecies differences in β‐ARs subtype distribution necessitate exploration. Propranolol at relatively low doses is unlikely to exert significant detrimental effects on testicular function, as the adverse effects reported in the literature generally occur at higher doses and for more extended administration periods.^[^ ^58^, ^59^, ^60^ ^]^ Thus, the protective effects observed in our model were likely attributable to the antagonism of stress‐induced β‐ARs activation. Including a propranolol‐only control group in future studies would help to clarify the independent effects of propranolol and enhance the translational value of our findings.

The canonical β‐ARs regulate cellular functions through the cAMP signaling pathway by activating transcription factors, notably CREB1.^[^ ^61^, ^62^ ^]^ Upon internalization, β‐ARs undergoes a G protein coupling switch characterized by diminished Gs binding and increased Gi association, inhibiting downstream cAMP/PKA signaling.^[^ ^7^, ^63^, ^64^ ^]^ This aligns with our findings that stress‐elevated NE promoted β‐arrestin1‐mediated β‐ARs internalization, subsequently upregulating Gi2 and downregulating CREB1. CREB1 has been found to regulate GPX4 expression.^[^ ^41^ ^]^ Reduced CREB1 expression sensitizes cells to ferroptosis.^[^ ^65^ ^]^ This study demonstrated that the internalization of β‐ARs inhibited the CREB1 and GPX4, a key mechanism of Sertoli cell ferroptosis caused by psychological stress. In addition, NE treatment also altered the expression of SLC7A11 and ACSL4 independently of CREB1, suggesting that NE may regulate ferroptosis in Sertoli cells through multiple parallel pathways. These results highlight the complexity of NE‐induced ferroptotic regulation and warrant further investigation to clarify the underlying molecular mechanisms.

In conclusion, our study identified psychological stress‐induced sympathetic hyperactivation as a key driver of male reproductive dysfunction, disrupting testicular redox homeostasis through β‐ARs hyperactivation driven by sustained NE elevation. β‐arrestin1‐dependent receptor desensitization led to Gi protein‐mediated suppression of the CREB1/GPX4 antioxidant pathway, triggering ferroptosis in Sertoli cells. These findings established a neuroendocrine‐ferroptosis axis linking neural stress responses to reproductive dysfunction. They suggested that targeted β‐ARs modulation may offer a potential strategy to preserve spermatogenesis under chronic stress. Although the number of Sertoli cells captured in the scRNA‐seq analysis was relatively low, our conclusions are supported by cellular and in vivo experiments, reinforcing the robustness of the findings. Validation in larger clinical cohorts and further mechanistic studies remains essential for future translation.

## 4 Experimental Section

### Psychological Stress Model and Drug Administration Protocols

Sprague‐Dawley (SD) rats (5–6 weeks) were purchased from the Laboratory Animal Center of Xi'an Jiaotong University. The rats were housed under specific pathogen‐free conditions with ad libitum access to standard food and water. They were maintained on a 12 h light or dark cycle at a temperature of 25 ± 2 °C and 50 ± 5% relative humidity. Psychological stress was induced using CUMS combined with social isolation. The CUMS protocol was adapted from previous studies with some modifications.^[^ ^66^ ^]^ The CUMS protocol consisted of the following seven mild stressors: an inverted light or dark cycle, cage tilting, forced swimming, damp sawdust, terrified‐sound stress, water deprivation, and food deprivation. One stressor was randomly applied each day for three consecutive weeks, with no repetition of the same stressor within the same week. The control animals were housed in separate rooms to prevent unintended exposure to stress.

The dosage of NE (HY‐13715B, MCE, USA) was based on previous research.^[^ ^67^, ^68^, ^69^ ^]^ NE was prepared by dissolving it in 0.1% ascorbic acid in sterile 0.9% NaCl solution and administered intraperitoneally at 1.5 mg kg^−1^ day^−1^ throughout the stress exposure period. Propranolol (HY‐B0573B, MCE, USA) was administered intraperitoneally at 2 mg kg^−1^ day^−1^,^[^ ^70^ ^]^ and Ferrostatin‐1 (HY‐100579, MCE, USA) at 1 mg kg^−1^ day^−1^.^[^ ^71^ ^]^ Both agents were injected 30 min before exposure to each stress. All rats were prospectively randomized into groups.

### Sucrose Preference Test

The sucrose preference test was used to assess anhedonia by measuring sucrose preference. Initially, the rats were trained for 24 h with two bottles containing 1% sucrose solution. This was followed by another 24 h period in which one bottle contained 1% sucrose and the other contained water, with the positions of the bottles switched to control for location bias. During testing, rats were fasted and deprived of water for 24 h. Sucrose and water consumption were measured over 12 h. The sucrose preference index was calculated by the proportion of sugar solution consumption.

### Sperm Density and DNA Fragmentation Assays

The cauda epididymidis were dissected and divided into three segments in a 24‐well plate containing 1.0 mL of preheated saline at 37 °C. The samples were gently shaken at 100 × g for 30 min to prepare the sperm suspension, which was then transferred to a Makler chamber to count individual spermatozoa under a light microscope. Sperm DNA fragmentation was assessed using a sperm nuclear integrity staining kit (Cellpro, Zhejiang, China) according to the manufacturer's instructions.

### Serum Collection and ELISA Assay

Human serum samples were obtained from psychologically stressed male infertile patients at the First Affiliated Hospital of Xi'an Jiaotong University. A total of 97 male participants who had experienced at least 12 months of unprotected intercourse without conception were enrolled. Psychological stress levels were assessed using the 14‐item Perceived Stress Scale (PSS‐14). Individuals with PSS‐14 scores ≥ 28 were categorized into the stress group (n = 66), while those with scores < 28 were classified into the control group (n = 31).^[^ ^72^ ^]^ Participants with any of the following conditions were excluded from the study: 1) Known chromosomal or genetic abnormalities; 2) Structural reproductive anomalies (e.g., varicocele or absence of vas deferens); 3) Urogenital infections (e.g., seminal vesiculitis, prostatitis, Chlamydia or Mycoplasma infections); 4) History of testicular injury, orchitis, or epididymitis; 5) Recent exposure to high temperature, radiation, or toxic chemicals; 6) Immunological causes such as antisperm antibodies.

Blood samples from male SD rats were clotted in tubes at room temperature and centrifuged at 4 °C for 20 min at 1500 × g.

Hormonal levels of CRH, ACTH, CORT, GC, GnRH, FSH, LH, T, and NE were measured using ELISA kits (Meibiao Biotech, Jiangsu, China) according to the manufacturer's instructions. The absorbance was measured using a microplate reader.

### H&E Staining

The testes were fixed, dehydrated using ethanol solutions, and embedded in paraffin. Paraffin blocks were then sectioned into 5 µm slices. The sections were dewaxed, hydrated, and stained with hematoxylin and eosin. Images were captured using the slide scanner system (Shenzhen Shengqiang Technology Co., Ltd., Teksqray SQS‐40X, China) and analyzed with Image J.

### Immunohistochemistry

Deparaffinization was performed using xylene, followed by hydration using an alcohol gradient. Antigen retrieval was performed using a citrate solution. Subsequent procedures were performed according to the guidelines provided by the immunohistochemical kit (ZSGB‐BIO, Beijing, China). Sertoli cells were labeled with WT1 (Abcam, ab267377, 1:50). Sertoli cells were counted in ten randomly selected circular spermatogenic tubules from each animal. Images were captured using an inverted microscope (Olympus, BX43, Japan) or a slide scanner system (Shenzhen Shengqiang Technology Co., Ltd., Teksqray SQS‐40X, China). Scale bars were calibrated and added to the respective image acquisition software.

### Clustering Analysis of Sertoli Cells in Single‐Cell Transcriptome

Initially, the cells were filtered based on UMI counts below 100000, with gene counts ranging from 200 to 3500. Cells with a mitochondrial content exceeding 5% were excluded. After filtering, data reduction and clustering were performed using Seurat v4.3.0.^[^ ^73^ ^]^ *NormalizeData* and *ScaleData* functions were applied to normalize and scale the gene expression data. Variable genes were selected using the *FindVariableFeatures* function, with the top 3000 most variable genes chosen for the principal component analysis (PCA). The batch effects between samples were removed using Harmony v1.1.0.^[^ ^74^ ^]^ Using the first 20 principal components, the cells were clustered into multiple groups using the *FindClusters* function. A total of 15 clusters were identified across six samples from the control and stress groups,^[^ ^29^ ^]^ with 486 Sertoli cells identified, of which 66 were from the control group and 420 were from the stress group. The FindMarkers function from the Seurat R package (v4.3.0) was employed to identify differentially expressed genes (DEGs) using the Wilcoxon Rank Sum test. DEGs were defined as genes with |log_2_Fold Change| > 1 and adjusted *p*‐value < 0.05. To explore their functional implications, GO and KEGG enrichment analyses were conducted using the clusterProfiler R package (v4.7.1.003), including all DEGs regardless of direction. scRNA‐seq data were available in the NIH Gene Expression Omnibus (GEO) under accession ID: GSE233950.

### TM4 Cell Treatments and Transfection

The Sertoli cell line, TM4, was obtained from Zhongqiaoxinzhou Biotech (Shanghai, China). TM4 cells were cultured in DMEM/F‐12 supplemented with 5% horse serum (Viva Cell, C2510, China), 2.5% fetal bovine serum (Dining, DC201, China), and 1% penicillin‐streptomycin (Gibco, C11330500BT, USA). The cells were maintained in a humidified incubator at 37 °C with 5% CO_2_.

The following concentrations of drugs were administered: NE (10 µM),^[^ ^67^, ^68^ ^]^ Atenolol (1 µM), ICI118551 (1 µM),^[^ ^75^, ^76^ ^]^ and Ferrostatin‐1 (0.5 µM).^[^ ^21^ ^]^ All drugs were purchased from MedChemExpress (USA). Where applicable, drugs were added simultaneously to ensure co‐treatment. *CREB1* overexpression vector and siRNAs were obtained from Jikai Biotechnology Co., Ltd (Shanghai, China) and Jiman Biotechnology Co., Ltd (Shanghai, China). The target sequences of the siRNAs are listed in Table S2 (Supporting Information). Cell transfection was conducted using JetPRIME reagent (Polyplus Transfection, France).

### Cell Viability Assay

TM4 cells were cultured in 96‐well plates and treated with the drugs at the designated concentrations for 24, 48, and 72 h. Following treatment, 10 µL of 3‐(4,5‐dimethylthiazol‐2‐yl)‐2,5‐diphenyltetrazolium bromide (MTT) reagent was added to each well. The plates were incubated at 37 °C for 4 h. The optical density (OD) was measured at 492 nm.

### Live‐Cell Analysis System

TM4 cells were seed into 96‐well plates and treated with the drugs. Cell counts were monitored every 6 h for 72 h using a Cytation 5 Cell Imaging Multi‐Mode Reader (Biotek, Winooski, VT, USA) with Gen5 Image software version 3.08.

### Cellular ROS and Lipid Peroxidation Detection

Cellular ROS levels were measured using 2,7‐dichlorofluorescein diacetate (DCFH‐DA) (Beyotime, S0033S, China). TM4 cells were cultured in 6‐well plates at a density of 1 × 10^5^ cells well^−1^. After the treatment, DCFH‐DA was diluted to the working concentration and added to each well. The cells were incubated at 37 °C for 30 min in the dark and analyzed by flow cytometry.

Lipid peroxidation was assessed by measuring the concentration of MDA) using a Lipid Peroxidation MDA Assay Kit (Jiancheng, A003‐1‐2, China) according to the manufacturer's instructions. The optical density (OD) was measured at 532 nm.

### Ferrous Iron Assay

The concentration of ferrous iron (Fe^2+^) was determined using a Ferrous Ion Content Assay Kit (BC5415; Solarbio) according to the manufacturer's instructions. The optical density (OD) was measured at 593 nm.

### Calcein‐AM/PI Staining

Calcein‐AM/PI staining was performed using a Calcein/PI Live/Dead Viability Assay Kit (Beyotime, C2015S, China). TM4 cells were incubated with 1× calcein‐AM and PI reagents at 37 °C for 30 min after 48 h of drug treatment. Images were captured using fluorescence microscopy (Nikon, Japan).

### Mitochondrial Membrane Potential (MMP) Assay

The MMP in TM4 cells was measured using a JC‐1 fluorescent probe (Yeasen, 40706ES60, China). Following treatment, cells were stained with 1× JC‐1 working solution for 20 min at 37 °C and then analyzed by flow cytometry. Mitochondrial membrane potential was quantified by calculating the red to green fluorescence ratio.

### Immunofluorescence Staining

Cells were fixed with 4% (v/v) paraformaldehyde for 15 min and permeabilized with 0.1% Triton X‐100 for 15 min, followed by blocking with 3% BSA for 1 h. Then, the cells were incubated with ACSL4 (Cat#A6826, ABconal), GPX4 (Cat#14432‐1‐Ig, ABconal), β‐tubulin (Cat#M20005, Abmart), β_1_‐AR (Cat#28323‐1‐Ig, Proteintech), β_2_‐AR (Cat# PTM‐6016, PTM BIO), EEA1 (Cat#68065‐1‐Ig, Proteintech) and LAMP1 (Cat# PTM‐5775, PTM BIO) overnight at 4 °C. After washing for three times with PBS, the cells were incubated with Alexa Fluor 488‐conjugated anti‐mouse IgG (Cat#AS037, Abconal), Alexa Fluor 594‐conjugated anti‐rabbit IgG (Cat#E‐AB‐1060, Elabscience), Alexa Fluor 488‐conjugated anti‐rabbit IgG (Cat#PC‐80013, PCM) and Alexa Fluor 594‐conjugated anti‐rabbit IgG (Cat#E‐AB‐1055, Elabscience) for 1 h. Subsequently, the slides were sealed with the antifade mounting medium with DAPI (P0131, Beyotime). Co‐localization images were obtained using a laser confocal microscope (Leica, Stellaris 5, Germany), and other images were obtained using a fluorescence microscope (ZEISS, Axio Vert.A1, Germany).

### RNA Isolation and Quantification

TM4 cells were lysed using TRIzol (GenStar, Shanghai, China) to extract total RNA, reverse‐transcribed using 5X All‐In‐One RT MasterMix (Yeasen, 11141ES, China). qRT‐PCR was performed on CFX ConnectTM Real‐Time System (Bio‐Rad, USA) using SYBR mixture (Yeasen, 11201ES, China). The fold difference was calculated using the 2^−ΔΔCt^ method with results normalized to GAPDH or β‐actin expression (Table S3, Supporting Information).

### Protein Extraction and Western Blot

Tissues or cells were homogenized in RIPA lysis buffer containing protease and phosphatase inhibitors to extract total proteins. Membrane proteins were isolated using the Cell Membrane/Plasma Protein Kit (FUDE, FD0198, China). Protein concentrations were determined using the BCA Protein Assay Kit (GenStar, Shanghai, China). Proteins were separated by SDS‐PAGE and transferred to PVDF membranes. After incubation with specific primary and secondary antibodies, the proteins were detected using the ChemiDoc Imaging System (Bio‐Rad, USA). Antibodies against ATP1A1(Cat# 14418‐1‐AP), β‐actin (Cat# 66009‐1‐Ig), β_1_‐AR (Cat# 66009‐1‐Ig), CREB1 (Cat# 12208‐1‐AP), GAPDH (Cat# 60004‐1‐Ig), Gi2 (Cat# 11136‐1‐AP), GPX4 (Cat# 14432‐1‐Ig), SLC7A11 (Cat# 26864‐1‐AP), TFR1 (Cat# 10084‐2‐AP), ZO‐1 (Cat# 21773‐1‐AP) were purchased from Proteintech (Wuhan, China). Antibody against ACSL4 (Cat# A6826) was obtained from Abclonal Technology (Wuhan, China). Antibodies against β‐arrestin1(Cat# PTM‐6445) and β_2_‐AR (Cat# PTM‐6016) were purchased from PTM BIO (Zhejiang, China). Antibodies against Claudin‐11 (Cat# CY2121) and SOX9 (Cat# CY5400) were obtained from Abways (Shanghai, China). The band intensity of the western blot results was quantified using Image J (NIH, USA).

### Animal Ethics

All animal experiments of this study were approved by the Animal Care Committee of Xi'an Jiaotong University (No. XJTULAC2022‐1151) and performed in strict accordance with the institutional guidelines for the care and use of laboratory animals.

### Human Ethics

The use of clinical data and serum samples in this study was approved by the Ethics Committee of the First Affiliated Hospital of Xi'an Jiaotong University (No. XJTU1AF2025LSYY‐650), with informed consent obtained from all participants.

### Statistical Analysis

All statistical analyses were performed using GraphPad Prism version 10.1.2. Data were presented as mean ± SEM. Before statistical testing, the normality of data distribution was assessed using the Shapiro–Wilk test, and the homogeneity of variances was evaluated using the *F*‐test (for two groups) or the Brown‐Forsythe test (for multiple groups). The unpaired two‐tailed Student's *t*‐test was used to compare the two groups. For comparisons involving more than two groups, one‐way ANOVA was performed, followed by Holm–Šídák's or Dunnett's multiple comparisons test, as appropriate. Two‐way ANOVA was conducted for experiments involving two independent variables. A *p*‐value of less than 0.05 was considered the threshold value for statistical significance. The following notations were used: ^*^ *p* < 0.05, ^**^ *p* < 0.01, and ^***^ *p* < 0.001.

## Conflict of Interest

The authors declare no conflict of interest.

## Author Contributions

L.Z. and S.G. contributed equally to this work. L.Z., S.G., X.X., and J.Y. performed in conceptualization. L.Z., S.G., R.L., and X.X. performed in data curation. L.Z., S.G., X.X., X.L., and R.L. performed in formal analysis. J.Y. performed in funding acquisition. L.Z., S.G., X.X., X.L., X.W., X.X., and Y.D. performed in investigation. L.Z., S.G., X.X., X.L., and X.W. performed in methodology. J.Y. performed in project administration. X.X., L.H., X.W., W.L., Y.D., and J.Y. performed in resources. R.L., L.H., X.W., and W.L. performed in software. L.H., Y.C., and J.Y. performed in supervision. L.Z., S.G., X.L., and X.W. performed in validation. L.Z. and R.L. performed in visualization. L.Z. and S.G. performed in writing–original draft. L.Z., S.G., Y.C., and J.Y. performed in writing–review and editing.

## Supporting information



Supporting Information

## Data Availability

The data that support the findings of this study are available from the corresponding author upon reasonable request.
