# Supplementary material for: Norepinephrine Induces Sertoli Cell Ferroptosis via Receptors Desensitization Causing Stress‐Related Male Reproductive Dysfunction
Source: Adv Sci (Weinh). 2025 Oct 6;12(48):e04817. doi: 10.1002/advs.202504817 (PMC12752569; doi:10.1002/advs.202504817)
Supplement: Supplementary file 1 — Supporting Information [file ADVS-12-e04817-s001.docx]

**Supplementary Figures and Tables**

**
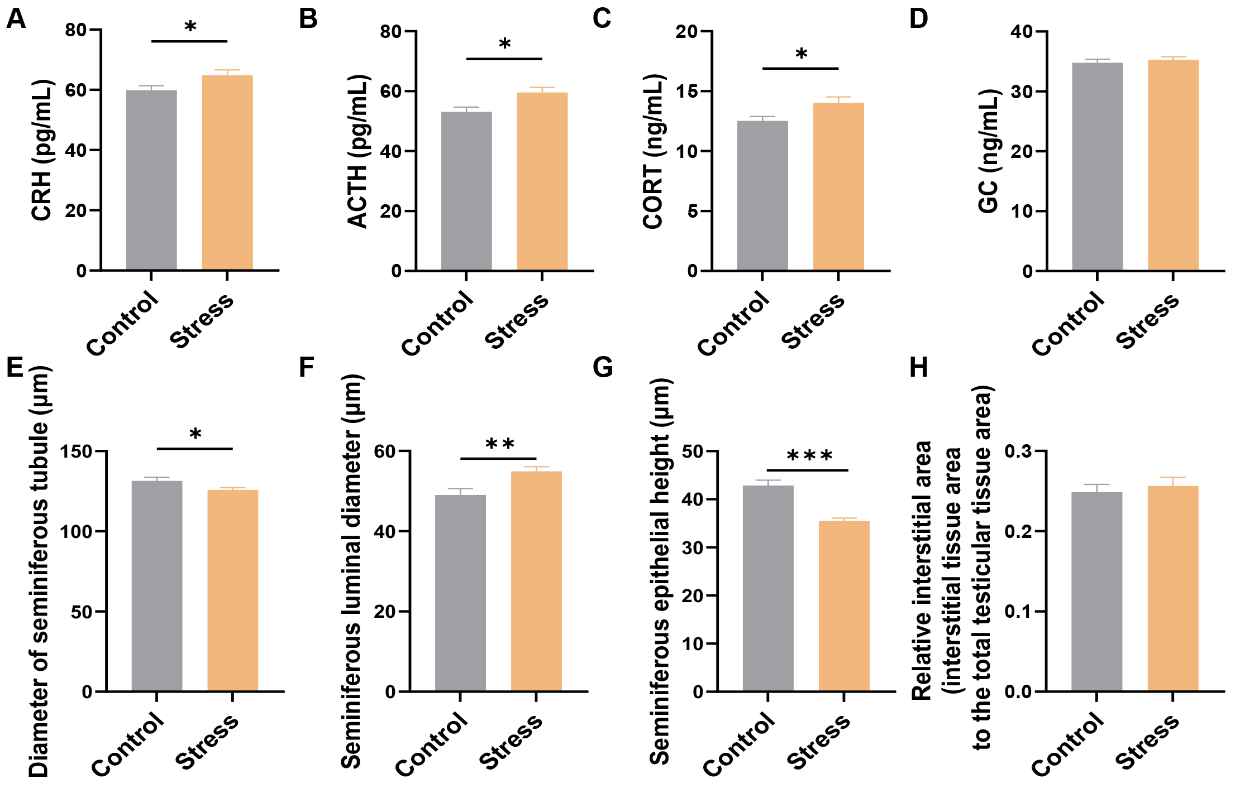
**

Figure S1. Psychological stress activates the hypothalamic-pituitary-adrenal (HPA) axis and impairs testicular structure.

(A-D), Serum levels of corticotropin-releasing hormone (CRH), adrenocorticotropic hormone (ACTH), corticosterone (CORT), and glucocorticoids (GC) in control and stressed rats (n = 10). (E-H), Morphometric analysis of testicular structure, including seminiferous tubule diameter, luminal diameter, epithelial height, and the ratio of interstitial area to total testicular area (n = 10). Data are presented as mean ± SEM. Statistical analysis was performed using an unpaired two-tailed Student’s *t*-test. **p* < 0.05, ***p*< 0.01, ****p* < 0.001.


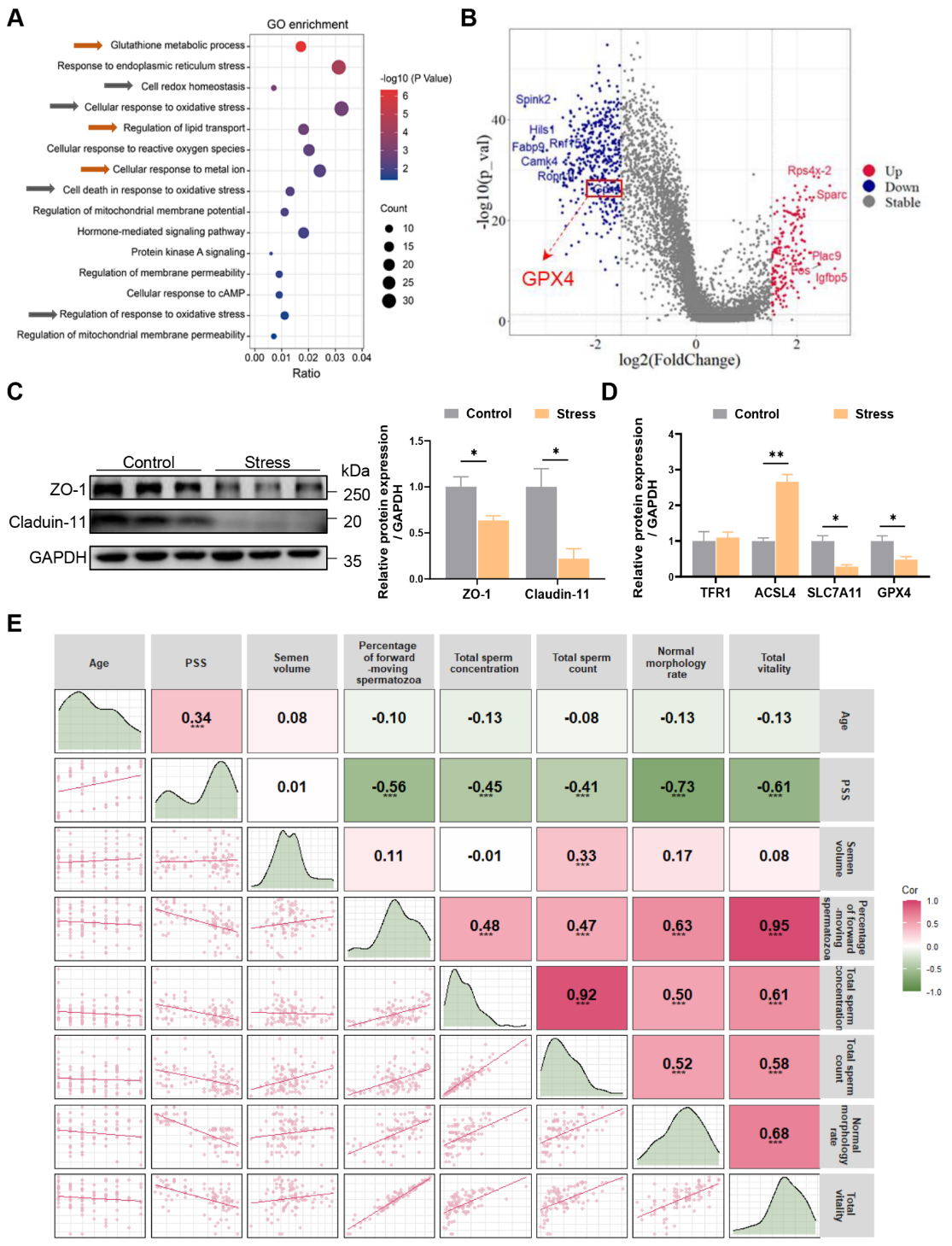


Figure S2. Psychological stress induces ferroptosis-related changes in Sertoli cells and impairs testicular barrier integrity.

(A), GO pathway enrichment analysis of scRNA-seq data from clustered Sertoli cells in the control and stress groups (n = 3). (B), Volcano plot showing downregulation of GPX4 expression in Sertoli cells under stress (n = 3). (C), Western blot analysis shows reduced expression of ZO-1 and Claudin-11 in testes (n = 3). (D), Quantification of TFR1, ACSL4, SLC7A11, and GPX4 protein levels in control and stressed testes (n = 3). (E), Spearman correlation matrix between psychological stress scores (PSS-14), age, and semen parameters. Color intensity indicates correlation strength and direction (red, positive; green, negative). All data are presented as mean ± SEM. Statistical analysis was performed using unpaired two-tailed Student’s *t*-test. **p* < 0.05; ***p* < 0.01.

**
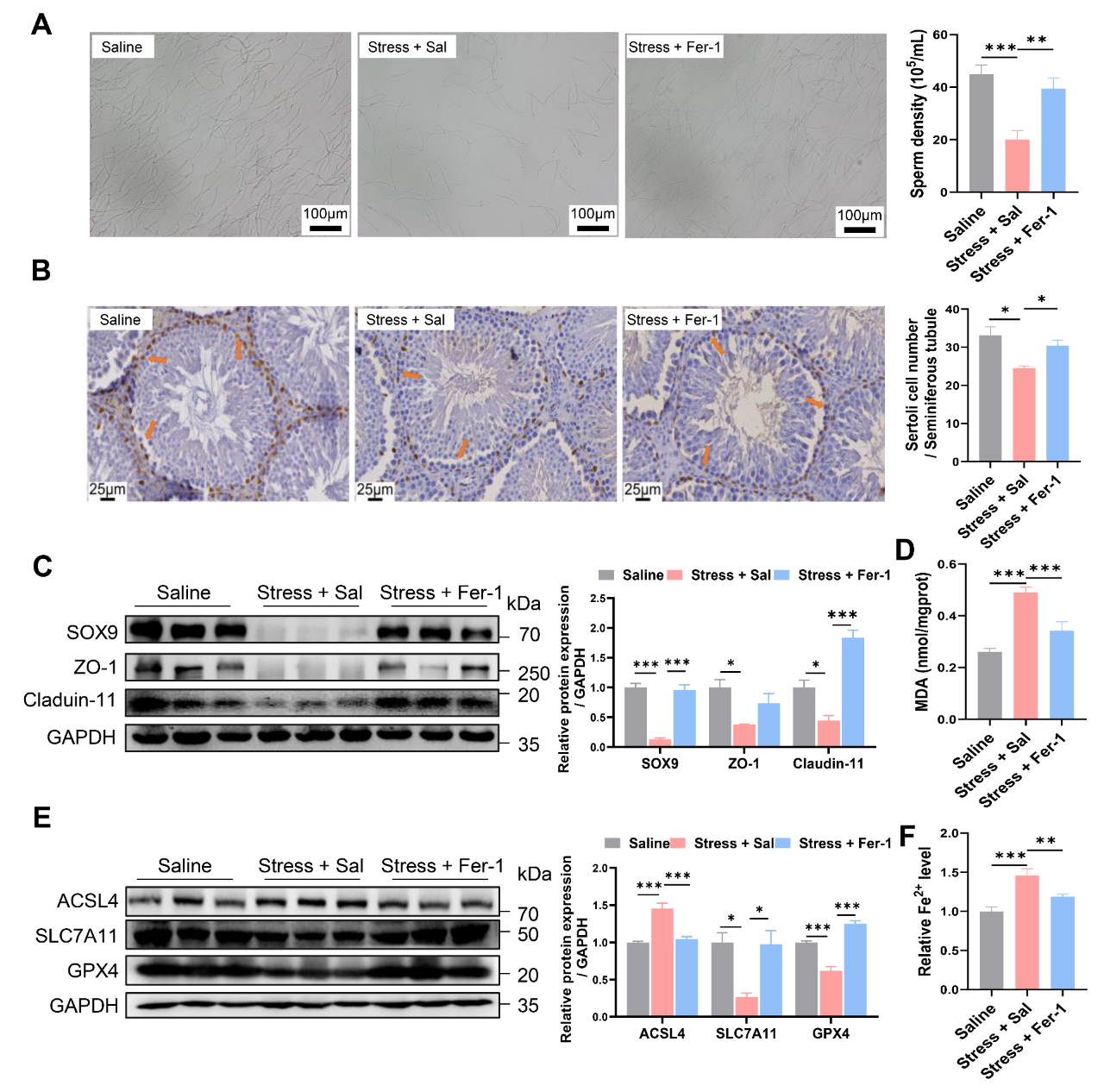
**

Figure S3. Ferroptosis inhibitor ameliorates stress-induced ferroptosis in Sertoli cells of male rats.

(A), Representative images showing sperm density in the cauda epididymis of experimental groups (scale bar, 100 µm, n = 6). (B), Ferroptosis inhibitor preventes the stress-induced reduction of Sertoli cell number in the testis (scale bar, 25 µm). Quantification of WT1-positive Sertoli cells per tubule (n = 6). (C), Western blot and quantification of SOX9, ZO-1, and Claudin-11 expression in testes (n = 3). (D), Quantification of MDA content in testes from three groups (n = 6). (E), Western blot and quantification of ACSL4, SLC7A11 and GPX4 in testes (n = 3). (F), Quantification of Fe^2+^ content in testes from three groups (n = 6). Data are presented as mean ± SEM. Statistical significance was assessed by one-way ANOVA with Holm–Šídák’s post hoc test. **p* < 0.05; ***p* < 0.01; ****p* < 0.001.

**
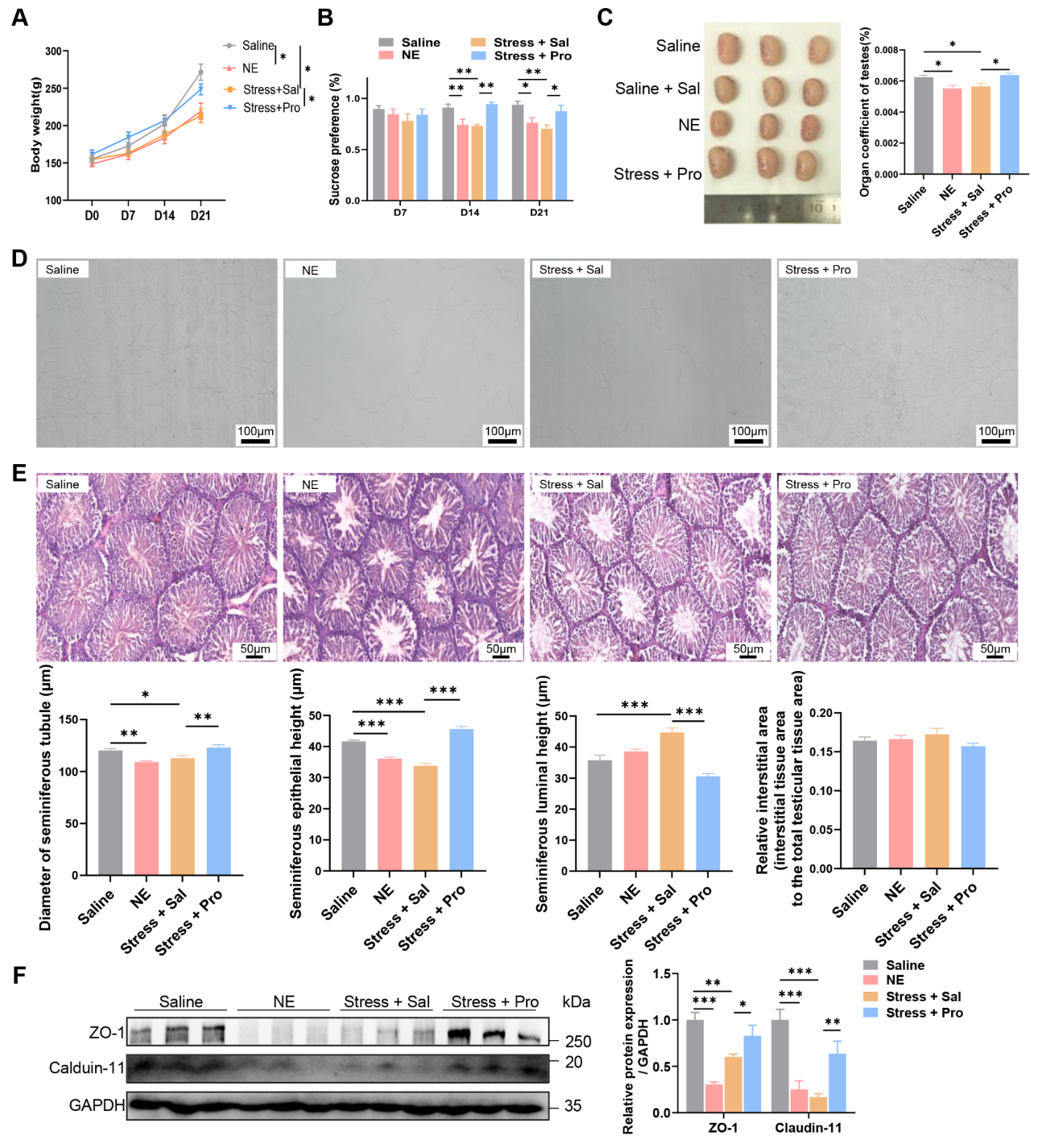
**

Figure S4. Psychological stress induces male reproductive dysfunction via NE-mediated β-adrenergic receptor activation.

(A), Body weight of experimental groups (n = 8). (B), Sucrose preference test (n = 8). (C), Representative testis images and organ coefficients for each group (n = 8). (D), Photomicrographs showing sperm density in the cauda epididymis (scale bar, 100 µm). (E), H&E staining and quantification of testicular tissue across four groups (scale bar, 50 µm, n = 8). (F), Western blot and quantification of ZO-1 and Claudin-11 expression in testes (n = 6). Data are presented as mean ± SEM. Statistical significance was assessed using two-way ANOVA or one-way ANOVA followed by Holm–Šídák’s post hoc test. **p* < 0.05; ***p* < 0.01; ****p* < 0.001.


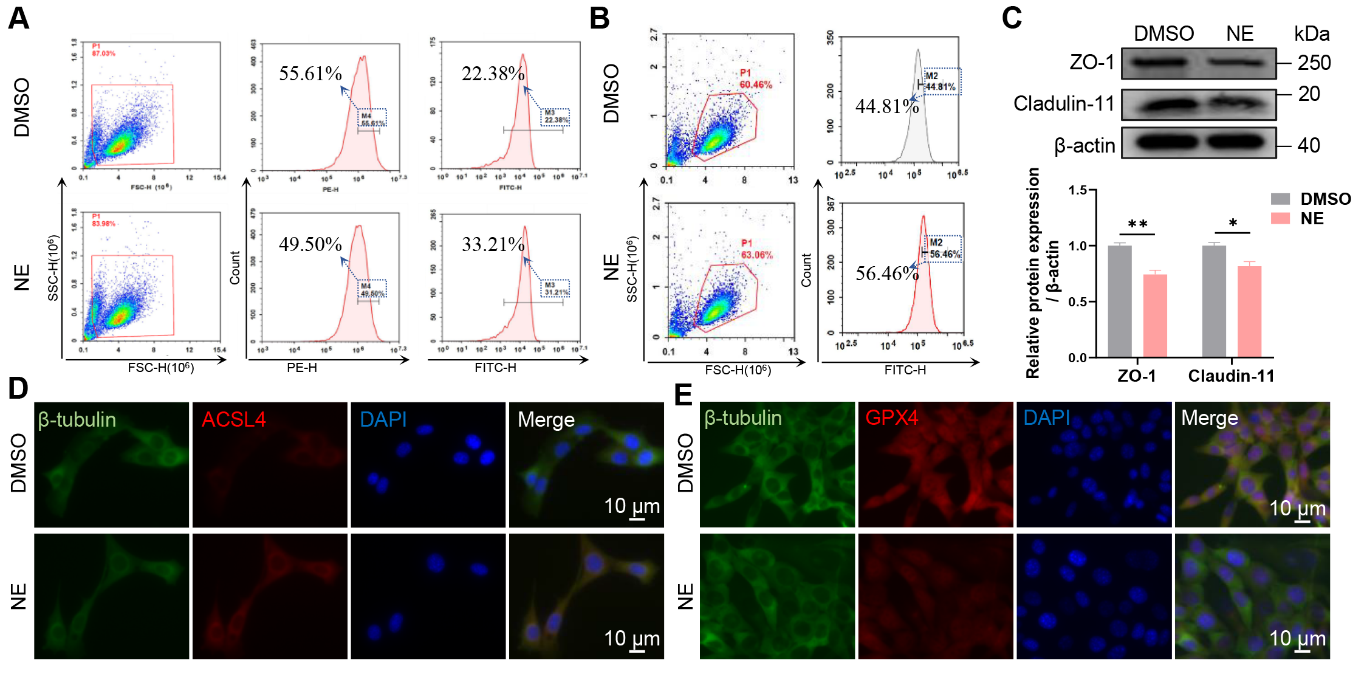


Figure S5. NE induces ferroptosis-related changes and tight junction disruption in Sertoli cells.

(A), Flow cytometry plots (SSC-H vs FSC-H, COUNT vs PE-H [red fluorescence], and COUNT vs FITC-H [green fluorescence]) from JC-1 staining, indicating changes in mitochondrial membrane potential. (B), Intracellular ROS levels measured using the DCFH-DA probe. (C), Western blot analysis demonstrating downregulation of tight junction proteins ZO-1 and Claudin-11 upon NE exposure (n = 3). (D-E), Immunofluorescence staining showing increased ACSL4 and decreased GPX4 intensities in Sertoli cells after NE treatment. Data are presented as mean ± SEM. Statistical significance was assessed using unpaired two-tailed Student’s *t*-test. **p* < 0.05; ***p* < 0.01.


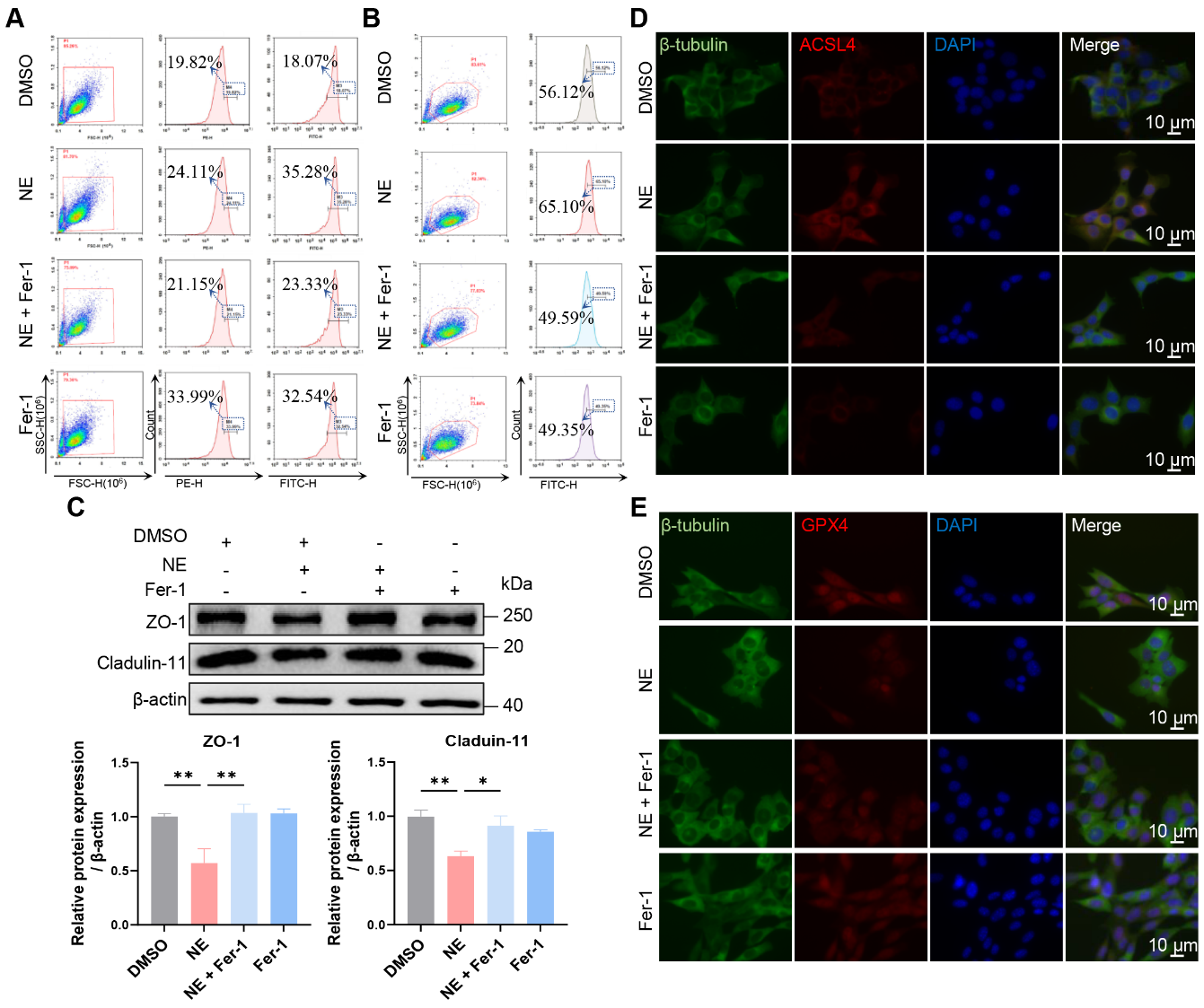


Figure S6. Ferroptosis inhibitor restores NE-induced ferroptosis and tight junction disruption in Sertoli cells.

(A), JC-1 staining plots illustrating that Fer-1 restored mitochondrial membrane potential impaired by NE treatment. (B) ROS detection plots showing that Fer-1 attenuated NE-induced ROS elevation. (C) Western blot analysis showed that Fer-1 reversed NE-induced downregulation of ZO-1 and Claudin-11 (n = 3). (D-E), Immunofluorescence staining demonstrated that Fer-1 treatment mitigated NE-induced upregulation of ACSL4 and downregulation of GPX4 in Sertoli cells. Data are shown as mean ± SEM. Statistical significance was assessed using one-way ANOVA followed by Dunnett post hoc test. **p*< 0.05; ***p* < 0.01.


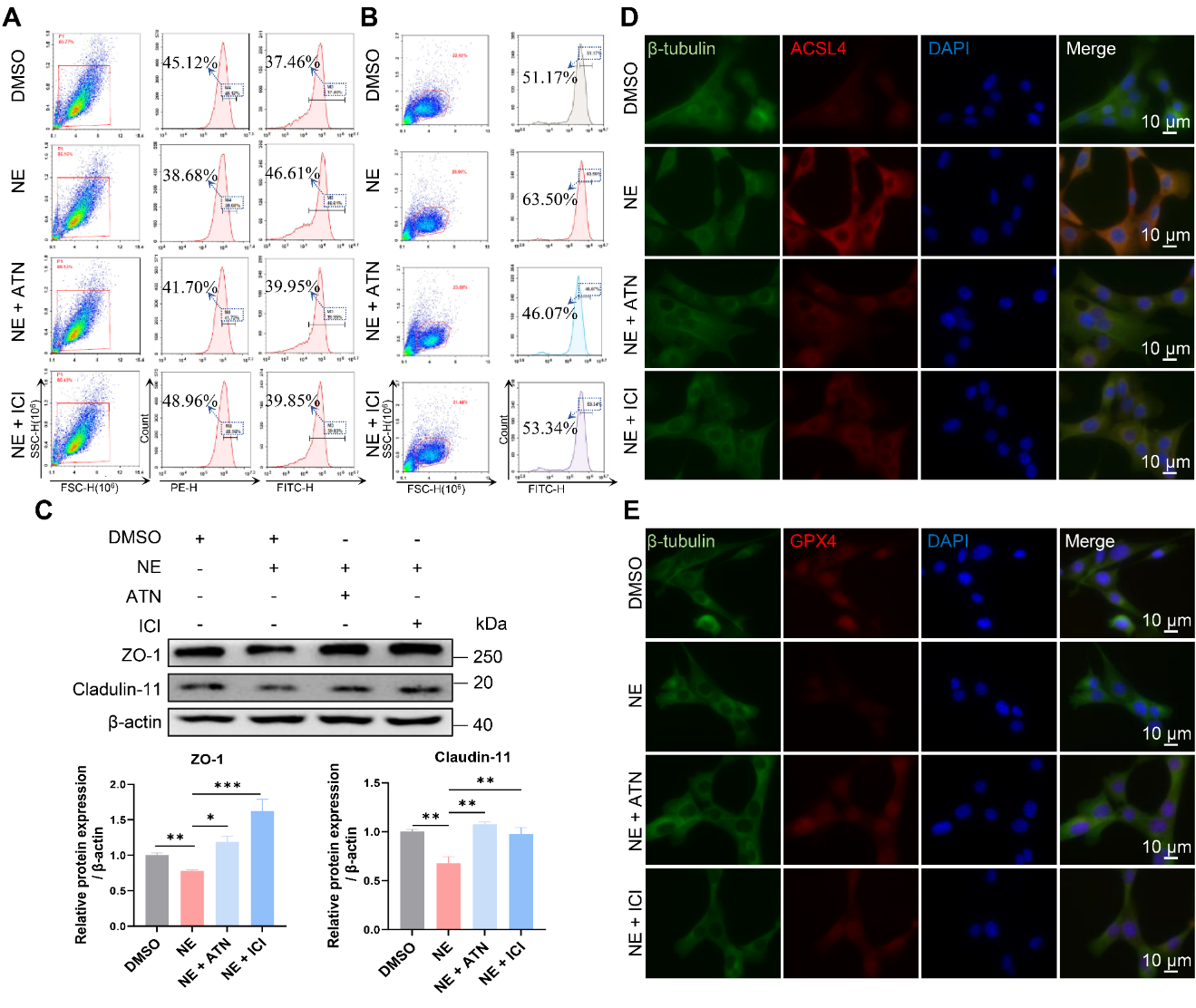


Figure S7. β-adrenergic receptor antagonists attenuate NE-induced ferroptosis and tight junction disruption in Sertoli cells.

(A), JC-1 staining plots illustrating the reversal of NE-induced mitochondrial membrane potential loss by Atenolol or ICI118551. (B), ROS detection plots showing that Atenolol or ICI118551 attenuated NE-induced ROS elevation. (C), Western blot showed both antagonists restored ZO-1 and Claudin-11 levels suppressed by NE (n = 3-4). (D–E), Immunofluorescence showed that Atenolol or ICI118551 reversed NE-induced ACSL4 increase and GPX4 decrease in Sertoli cells. Data are shown as mean ± SEM. Statistical significance was assessed using one-way ANOVA followed by Dunnett post hoc test. **p* < 0.05; ***p* < 0.01; ****p* < 0.001.


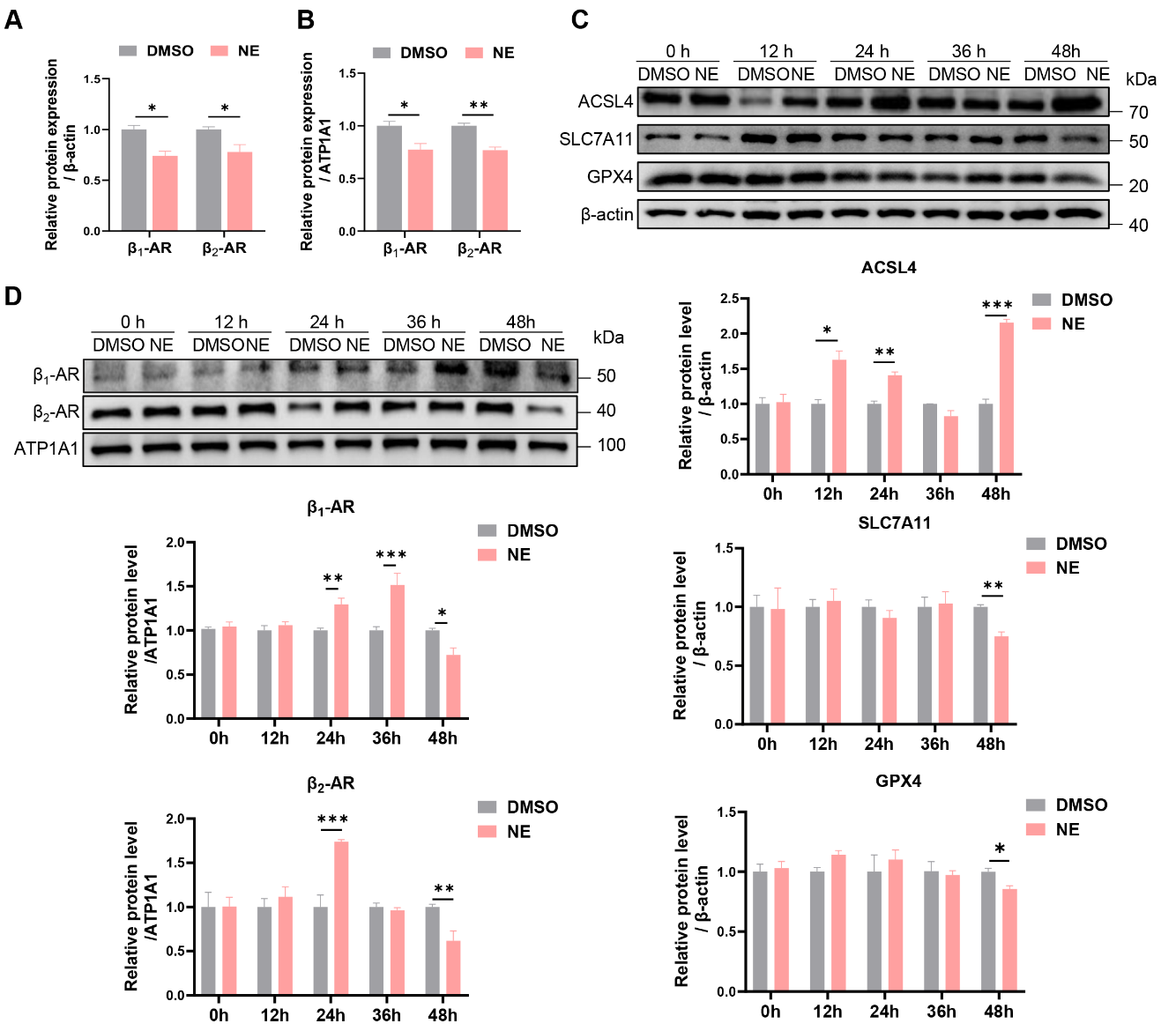


Figure S8. β_1_ – and β_2_-AR internalization and downregulation mediate NE-induced ferroptosis in Sertoli cells.

(A), Quantification of total β_1_-AR and β_2_-AR protein after 48 h of NE treatment (n = 3). (B), Quantification of membrane-localized β_1_-AR and β_2_-AR following 48 h of NE exposure (n = 3-4). (C) Time-course western blot analysis of ferroptosis-related proteins ACSL4, SLC7A11, and GPX4 at 0, 12, 24, 36, and 48 h post-NE treatment (n = 3). (D) Time-course western blot analysis of β_1_-AR and β_2_-AR expression following NE treatment, normalized to ATP1A1 (n = 3-4). Data are shown as mean ± SEM. Statistical analysis was performed by unpaired two-tailed Student’s *t*-test. **p* < 0.05; ***p* < 0.01; ****p* < 0.001


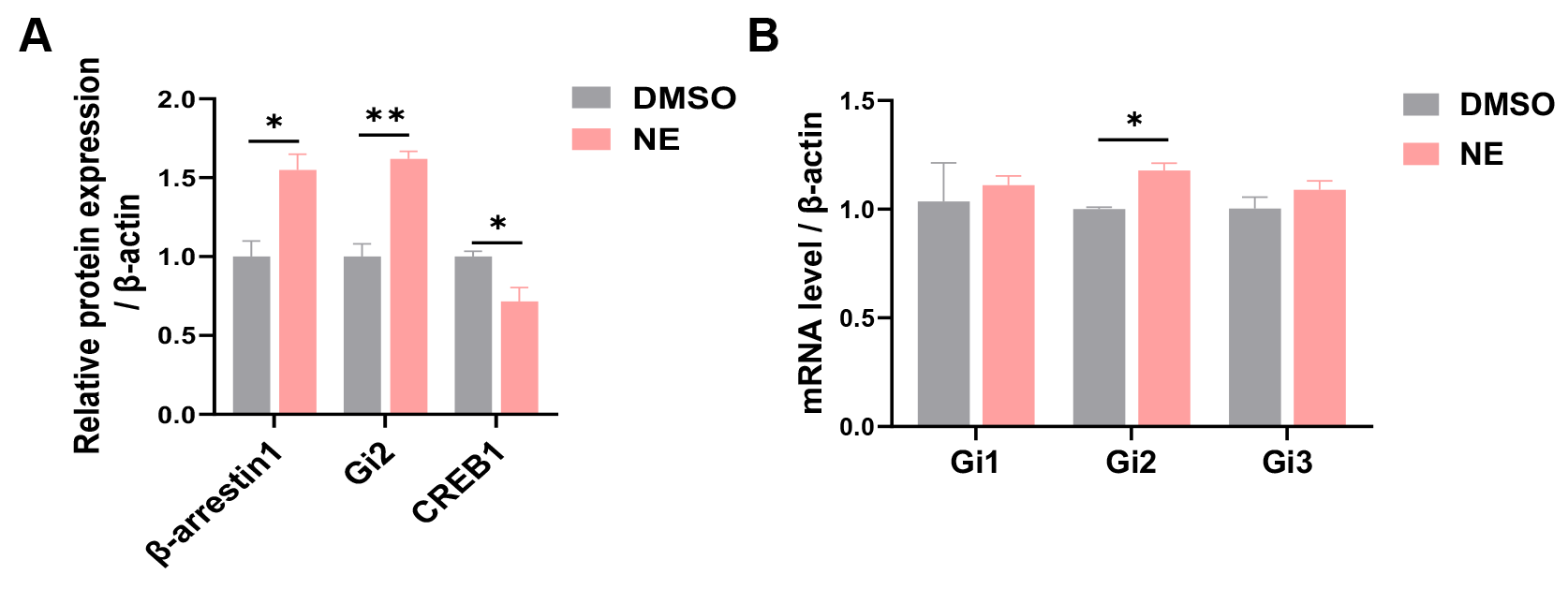


Figure S9. NE upregulates β-arrestin1 and Gi2 expression while downregulating CREB1 in Sertoli cells.

(A), Quantification of β-arrestin1, Gi2 and CREB1 protein levels after NE treatment (n = 3). (B), mRNA levels of inhibitory G proteins following NE treatment (n = 3). Data are shown as mean ± SEM. Statistical analysis was performed by unpaired two-tailed Student’s *t*-test. **p* < 0.05; ***p* < 0.01.


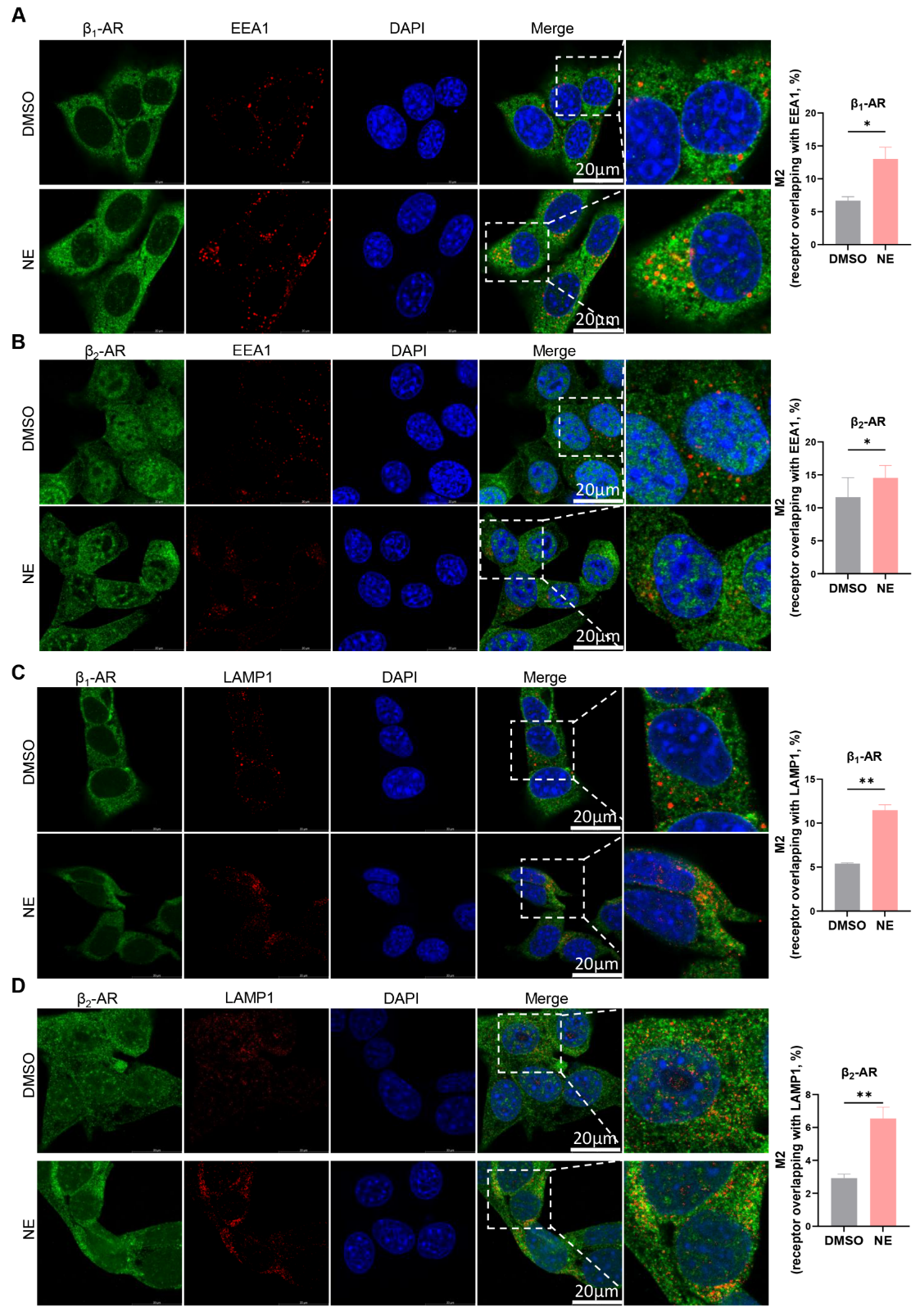


Figure S10. Co-localization analysis of β-adrenergic receptors with endosomal and lysosomal markers in Sertoli cells following NE treatment.

(A-B), Co-localization of β_1_-AR and β_2_-AR with early endosome marker EEA1 after 24 h NE treatment. (C-D), Co-localization of β_1_-AR and β_2_-AR with lysosomal marker LAMP1 after 48 h NE treatment. Co-localization was quantified using Manders’ overlap coefficient M2, representing the proportion of receptor signal overlapping with the marker. Higher M2 values indicate stronger co-localization. Data are presented as mean ± SEM. Statistical analysis by unpaired two-tailed Student’s *t*-test. *p < 0.05; **p < 0.01.


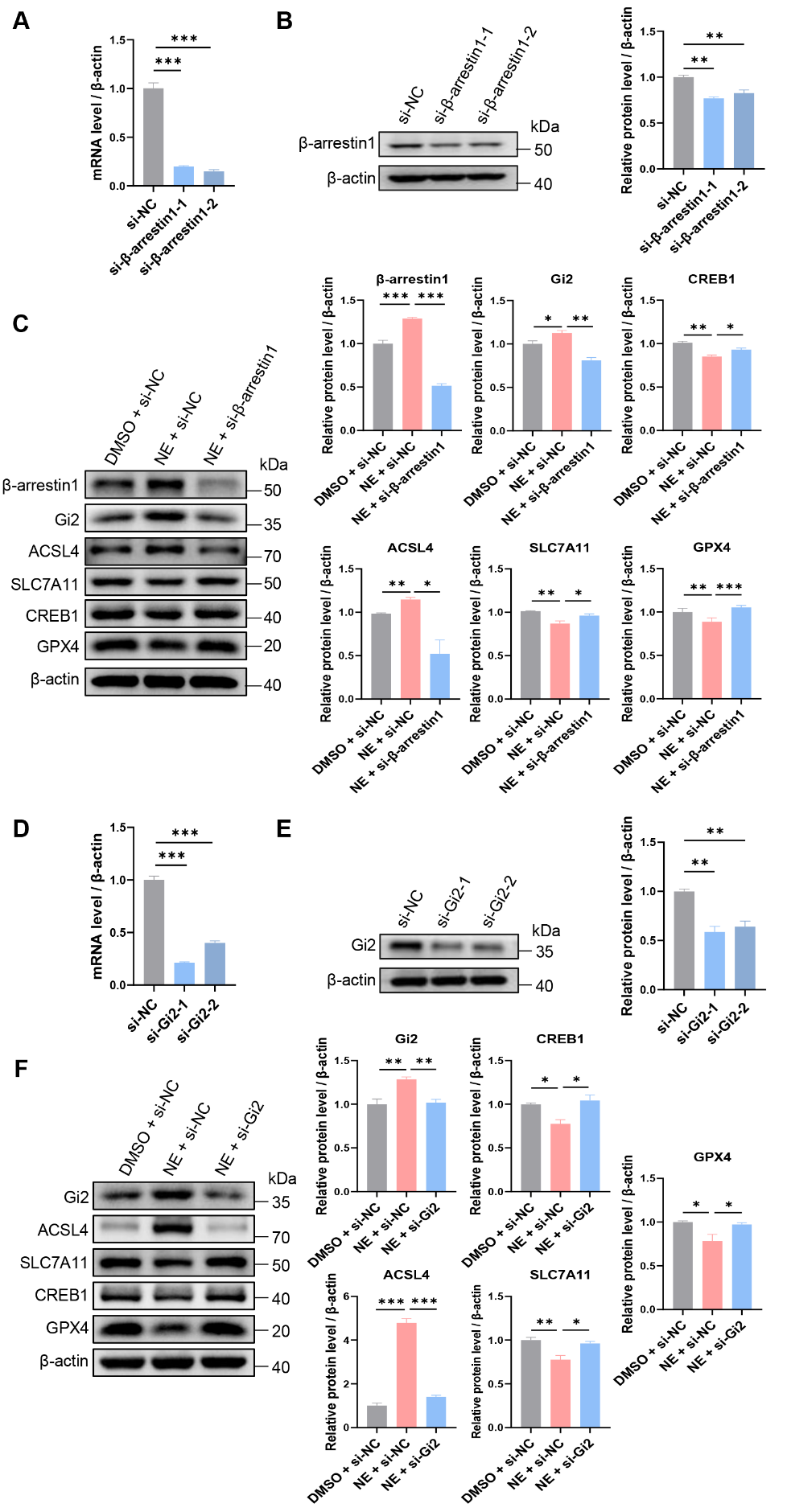


Figure S11. Effects of β-arrestin1 and Gi2 knockdown on NE-induced protein expression changes in Sertoli cells.

(A), mRNA levels of β-arrestin1 after siRNA transfection showing effective knockdown. (B), Western blot analysis confirming β-arrestin1 protein knockdown (n = 3). (C), si-β-arrestin1 reverses NE-induced upregulation of β-arrestin1, Gi2, and ACSL4, and downregulation of CREB1, SLC7A11, and GPX4 (n = 3). (D), mRNA levels of Gi2 after siRNA transfection, showing effective knockdown (n = 3). (E), Western blot analysis confirming Gi2 protein knockdown (n = 3). (F), si-Gi2 reversed NE-induced upregulation of Gi2 and ACSL4, and downregulation of CREB1, SLC7A11, and GPX4 (n = 3). Data are expressed as mean ± SEM. Statistical analysis by one-way ANOVA followed by Dunnett post hoc test. *p < 0.05; **p < 0.01; ***p < 0.001.


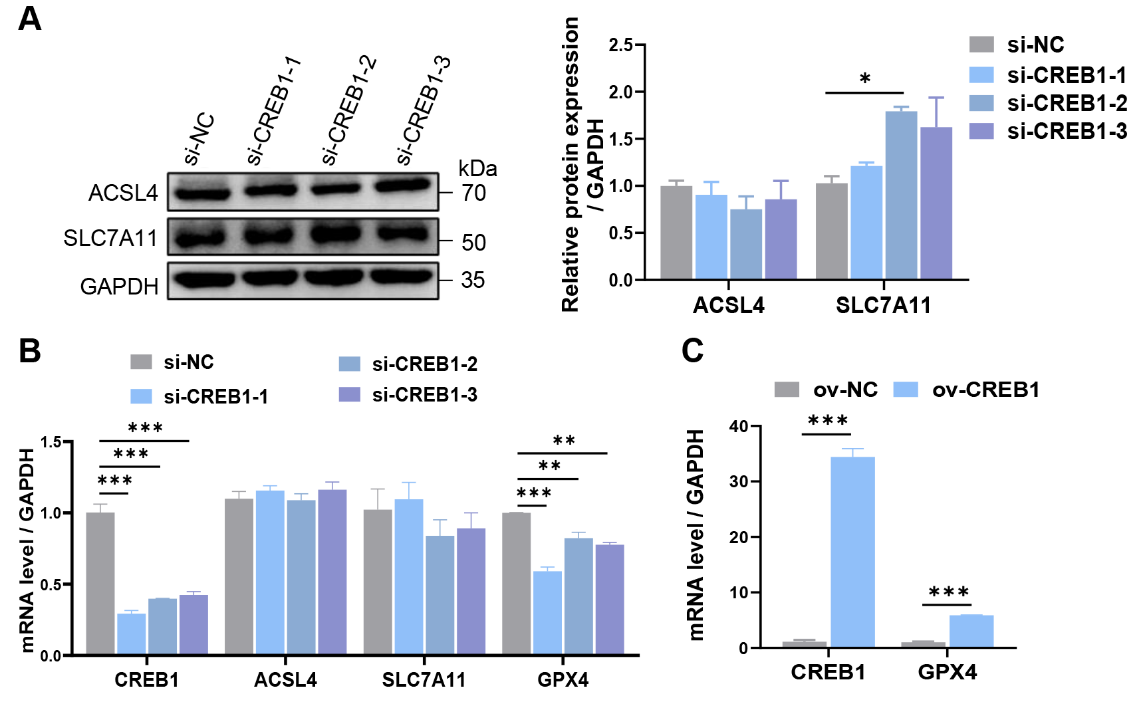


Figure S12. CREB1 regulates GPX4 expression in Sertoli cells.

(A), Western blot and quantification of ACSL4 and SLC7A11 after si*CREB1* treatment (n = 3). (B), mRNA levels of CREB1, ACSL4, SLC7A11, and GPX4 after si*CREB1* treatment (n = 3). (C), mRNA levels of CREB1 and GPX4 after *CREB1* overexpression (n = 3). Data are expressed as mean ± SEM. Statistical analysis was performed by unpaired two-tailed Student’s *t*-test or one-way ANOVA followed by Dunnett post hoc test. **p* < 0.05; ***p* < 0.01; ****p* < 0.001.

**Supplementary Table 1. Demographic and clinical characteristics of the study participants**

| Characteristics | Stress group(n=66) | Control group(n=31) | *p*-value |
| --- | --- | --- | --- |
| Age (years) | 31.94±2.07 | 31.68±2.27 | 0.5347 |
| PSS score (0-56) | 39.65±4.10 | 21.42±3.42 | <0.0001 |
| Semen volume (mL) | 3.68±1.20 | 3.60±0.91 | 0.7918 |
| Percentage of forward-moving spermatozoa (%) | 46.18±17.22 | 66.59±14.87 | <0.0001 |
| Total sperm concentration (×10⁶/mL) | 55.65±34.18 | 108.49±62.10 | <0.0001 |
| Total sperm count (×10⁶) | 209.01±145.97 | 376.55±209.50 | 0.0002 |
| Normal morphology rate (%) | 3.26±0.57 | 4.49±0.29 | <0.0001 |

**Supplementary Table 2.** Sequences of siRNAs used in this study

|  | sense（5'-3'） | antisense（5'-3'） |
| --- | --- | --- |
| siCREB1-1 | GUCCGUCUAAUGAAGAACATT | UGUUCUUCAUUAGACGGACTT |
| siCREB1-2 | CAACCAAGUUGUUGUUCAATT | UUGAACAACAACUUGGUUGTT |
| siCREB1-3 | GGCUAACAAUGGUACGGAUTT | AUCCGUACCAUUGUUAGCCTT |
| si-β-arrestin1-1 | GGCCUGUGGUGUGGAUUAU | AUAAUCCACACCACAGGCC |
| si-β-arrestin1-2 | AGCCUUCUGUGCUGAGAAC | GUUCUCAGCACAGAAGGCU |
| si-Gi2-1 | CAGAGUGACUACAUCCCUA | UAGGGAUGUAGUCACUCUG |
| si-Gi2-1 | CCAUCGUCAAGCAGAUGAA | UUCAUCUGCUUGACGAUGG |

**Supplement Table 3**. Primer sequences used for quantitative real-time PCR (qRT-PCR)

| Target gene | Species | -(F) | -(R) |
| --- | --- | --- | --- |
| β_1_-AR | Mouse | ACTGTGGACAGCGATTCGAG | CGGGGCTTTTCTGTACGTCT |
| β_2_-AR | Mouse | TGGTGGTGATGGTCTTTGTC | GTCTTGAGGGCTTTGTGCTC |
| β-arrestin1 | Mouse | GATCCCGCCAAACCTTCCAT | CAGGGGCATACTGAACCTTCC |
| Gi1 | Mouse | TTGGTTCTTTGTCTGGCAGTT | CAGGGTAAGGGCAGTGACATT |
| Gi2 | Mouse | GCTCAATGACTCAGCCGCTT | CACGATGCCCGTGGTCTTCA |
| Gi3 | Mouse | TAGCAGGTCCAGGGAATATC | GGGTCTCCACAATGCCTGTA |
| CREB1 | Mouse | GCTCCCACTGTAACCTTAGTG | GGACTTGTGGAGACTGGATAAC |
| GPX4 | Mouse | CAGGAGCCAGGAAGTAAT | CAGCCGTTCTTATCAATGAG |
| SLC7A11 | Mouse | GGCACCGTCATCGGATCAG | CTCCACAGGCAGACCAGAAAA |
| ACSL4 | Mouse | CTCACCATTATATTGCTGCCTGT | TCTCTTTGCCATAGCGTTTTTCT |
| β-actin | Mouse | TCTGGCACCACACCTTCTACAA | TTTTCACGGTTGGCCTTAGG |
| GAPDH | Mouse | TGTGTCCGTCGTGGATCTGA | CCTGCTTCACCACCTTCTTGA |
